# Visible‐Light Sensitized Isomerization in the Lipid Bilayer Enables Activation of a Transmembrane Transporter

**DOI:** 10.1002/anie.6015167

**Published:** 2026-04-05

**Authors:** Julia Villalva, Abhishek Mondal, Willem Marulanda, Jasper E. Bos, Sylvestre Bonnet, Anjali Pandit, Sander J. Wezenberg

**Affiliations:** ^1^ Leiden Institute of Chemistry Leiden University CC Leiden The Netherlands

**Keywords:** anion transport, lipid bilayers, molecular switches, photosensitization, stiff‐stilbene

## Abstract

Currently, there is major interest in embedding light‐responsive (supra)molecular systems in phospholipid bilayer membranes, for example, to control transmembrane transport processes. Most of these systems are operated by harmful UV‐light. Although in solution phase the isomerization of photoswitchable systems has been achieved using visible‐light‐absorbing photosensitizers, no cases are known yet of sensitized isomerization in a lipid bilayer environment. Herein, we show that the active isomer of a stiff‐stilbene based chloride transporter can be generated in situ by irradiation and energy transfer from a ruthenium tris(bipyridyl) complex. In the bilayer, this process is more efficient than in solution, as a benefit of the higher effective concentration of sensitizer and transporter upon confinement. Our work offers an approach to induce isomerization of membrane‐embedded systems by visible light, without the need of synthetic modification and loss of function or activity.

## Graphical abstract

A stiff‐stilbene‐based transmembrane anion transporter is isomerized by energy transfer from a ruthenium tris(bipyridyl) photosensitizer in the lipid bilayer. This sensitized isomerization allows activation of chloride transport using visible instead of UV light, without the need to chemically modify the stiff‐stilbene photoswitch.

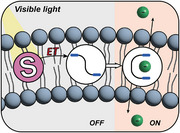

## 1 Introduction

Synthetic systems based on light‐driven molecular switches and motors are increasingly embedded in phospholipid bilayer membranes, in particular to control ion transport processes [1, 2, 3, 4, 5, 6, 7, 8, 9, 10, 11, 12, 13, 14, 15, 16, 17]. The vast majority of these systems relies on the use of harmful high‐energy UV light. The scarce examples that allow operation by visible light have structurally modified azo‐switching units with lowered HOMO‐LUMO gaps [6, 7, 10, 12]. However, structural adaptation of known transporters poses synthetic challenges and could compromise activity. Alternative strategies towards visible‐light switching that do not require synthetic modifications are therefore highly desired.

In addition to chemical derivatization, energy transfer from photosensitizers (PSs) to molecular switches and motors has been used to enable isomerization by visible light [18, 19, 20, 21, 22, 23]. Here, metal complexes have commonly been employed as triplet sensitizers [24, 25, 26, 27, 28, 29, 30, 31, 32, 33]. Excitation of their metal‐to‐ligand charge transfer (^1^MLCT) band followed by intersystem crossing can result in energy transfer and hence isomerization from the triplet state of a photoswitch. Yet, current sensitization studies have been performed in solution, while triplet energy transfer relies on collisions between molecules, depending on diffusion rates. Supramolecular encapsulation could enhance the sensitization process by increasing the effective concentration of sensitizer and photoswitch within a confined space. Indeed, enhancement of the sensitization efficiency for isomerization of azobenzene and dithienylethene was shown *via* confinement in a coordination cage [34] and micelle [35], respectively. However, despite the progress in the development of light‐activated membrane‐embedded systems [1, 2, 3, 4, 5, 6, 7, 8, 9, 10, 11, 12, 13, 14, 15, 16, 17], sensitized isomerization has—to our knowledge—not been demonstrated in phospholipid bilayers.

**SCHEME 1:**
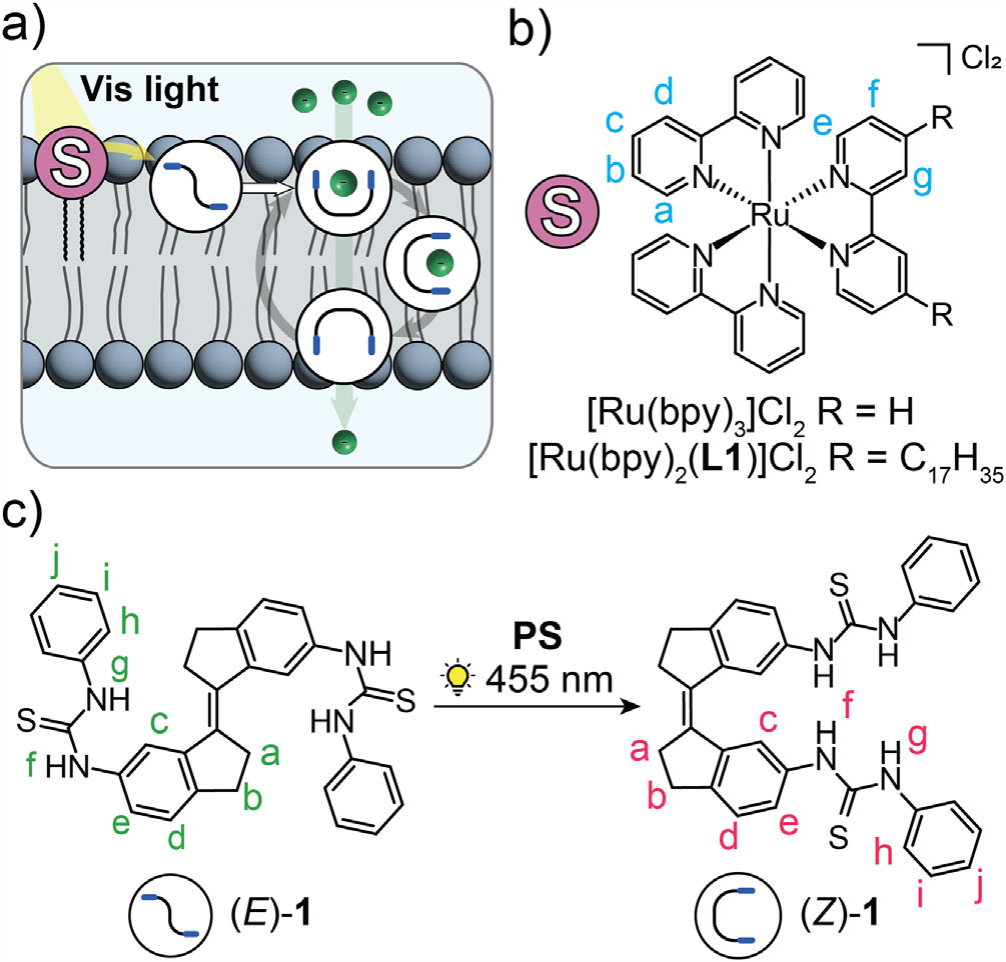
(a) Schematic representation of energy transfer in the lipid bilayer to activate transmembrane transport of chloride (green spheres). Structures of (b) photosensitizers and (c) transporter isomers involved.

Previously, our group developed stiff‐stilbene bis‐thiourea **1** (Scheme 1) [36], of which the *Z*‐isomer displayed a nearly 30‐times higher transmembrane chloride transport activity than the *E*‐isomer [11]. These isomers were interconverted by 365/385 nm UV light, allowing in situ (de)activation of chloride transport across the lipid bilayer membrane. The use of stiff‐stilbene as backbone of photoswitchable anion transporters has proven advantageous due to its high thermal stability and structural rigidity, as well as the large difference in geometry between isomers [1, 37, 38]. On the other hand, a drawback is that it needs to be operated with UV light. While we and others have addressed this issue by introducing electron‐donating and ‐withdrawing groups in *para*‐position with respect to the double bond to enable visible‐light‐excitation [39, 40, 41], applying this strategy to transporter **1** is synthetically demanding and would reduce its thermal stability. We therefore set out to investigate whether *E*→*Z* isomerization of this compound—and with that activation of transmembrane transport—can be mediated by a photosensitizer. Among available sensitizers, the tris(2,2'‐bipyridine)ruthenium(II) complex was deemed suitable for this study because of its high‐energy triplet excited state [42]. Further, this complex was used earlier to isomerize stilbene [24, 30, 43] and overcrowded alkene [44, 45] using visible light (∼450 nm), while it should be noted that sensitized isomerization of structurally‐related stiff‐stilbene has not been reported before.

We here show sensitized isomerization of stiff‐stilbene based transporter **1** in solution and in a lipid bilayer using ruthenium bipyridyl complexes [Ru(bpy)_3_]Cl_2_ and [Ru(bpy)_2_(**L1**)]Cl_2_ (see Scheme 1). The latter alkylated complex is required for the studies in the lipid bilayer, showing an enhancement of the energy transfer process compared to homogeneous solution studies. Finally, this sensitization strategy is applied to activate transmembrane transport of chloride by visible light (455 and 525 nm). We expect this approach to be applicable to other membrane‐embedded systems responsive to light, avoiding the need for UV excitation and therefore, bringing practical applications closer to reality. In addition, we foresee that asymmetric insertion of the photosensitizer in the membrane could enable active ion transport (against a concentration gradient) [46, 47].

## 2 Results and Discussion

**FIGURE 1:**
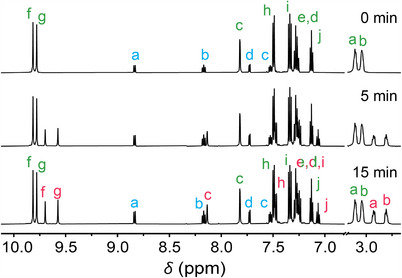
^1^H NMR spectra (500 MHz, 293 K) of (*E*)‐**1** (2.0 mM) and [Ru(bpy)_3_)]Cl_2_ (0.2 mM) in DMSO‐*d_6_* before and after irradiation with 455 nm light (24.5 mW). For the lettering assignment, see Scheme 1.

The possibility of sensitizing the isomerization of stiff‐stilbene based transporter **1** was initially studied by ^1^H NMR spectroscopy. As the solvent, DMSO‐*d*_6_ was chosen to ensure the full dissolution of both isomers [36]. As expected, visible‐light irradiation (*λ*_irr_ = 455 nm) of a solution of (*E*)‐**1** in the absence of photosensitizer did not lead to any changes in the ^1^H NMR spectrum (Figure S1). Gratifyingly, when 0.1 equiv. of [Ru(bpy)_3_]Cl_2_ was present, irradiation with 455 nm light (*i.e*. into the MLCT absorption band) gave a new set of signals that belonged to (*Z*)‐**1** (Figures 1, S2, and S3). Irradiation was stopped when the relative *E*/*Z* integral ratio did not change further, revealing that the photostationary state (PSS_PS_) had been reached. The final (*E*/*Z*) ratio obtained was 68:32, which was different from the previously reported ratio 53:47 observed upon direct UV excitation (*λ*_irr_ = 365 nm) [11]. Photoconversion to the *Z*‐isomer by this sensitization approach is thus slightly lower than with UV irradiation, which was also observed for overcrowded alkene‐based rotary motors [27, 45], and is most likely ascribed to the triplet‐ instead of singlet‐state involvement in the isomerization process.

This photoisomerization process was additionally followed by UV–vis spectroscopy. The absorption spectrum of a sample of (*E*)‐**1** with 0.1 equiv. of [Ru(bpy)_3_]Cl_2_ in DMSO showed the characteristic maxima for the stiff‐stilbene *E*‐isomer at *λ*_max_ = 345 and 360 nm as well as the MLCT absorption band of the ruthenium complex around 455 nm (Figure S8). Irradiation with 455 nm light led to a decrease in UV absorbance maxima, whereas the absorption between *λ* = 370–400 nm slightly increased. Further, a clear isosbestic point was found at *λ* = 370 nm. These UV–vis spectral changes are indicative of unimolecular *E*→*Z* isomerization of stiff‐stilbene based bis‐thiourea **1** [11, 36], in line with the observations by ^1^H NMR spectroscopy described above.

**FIGURE 2:**
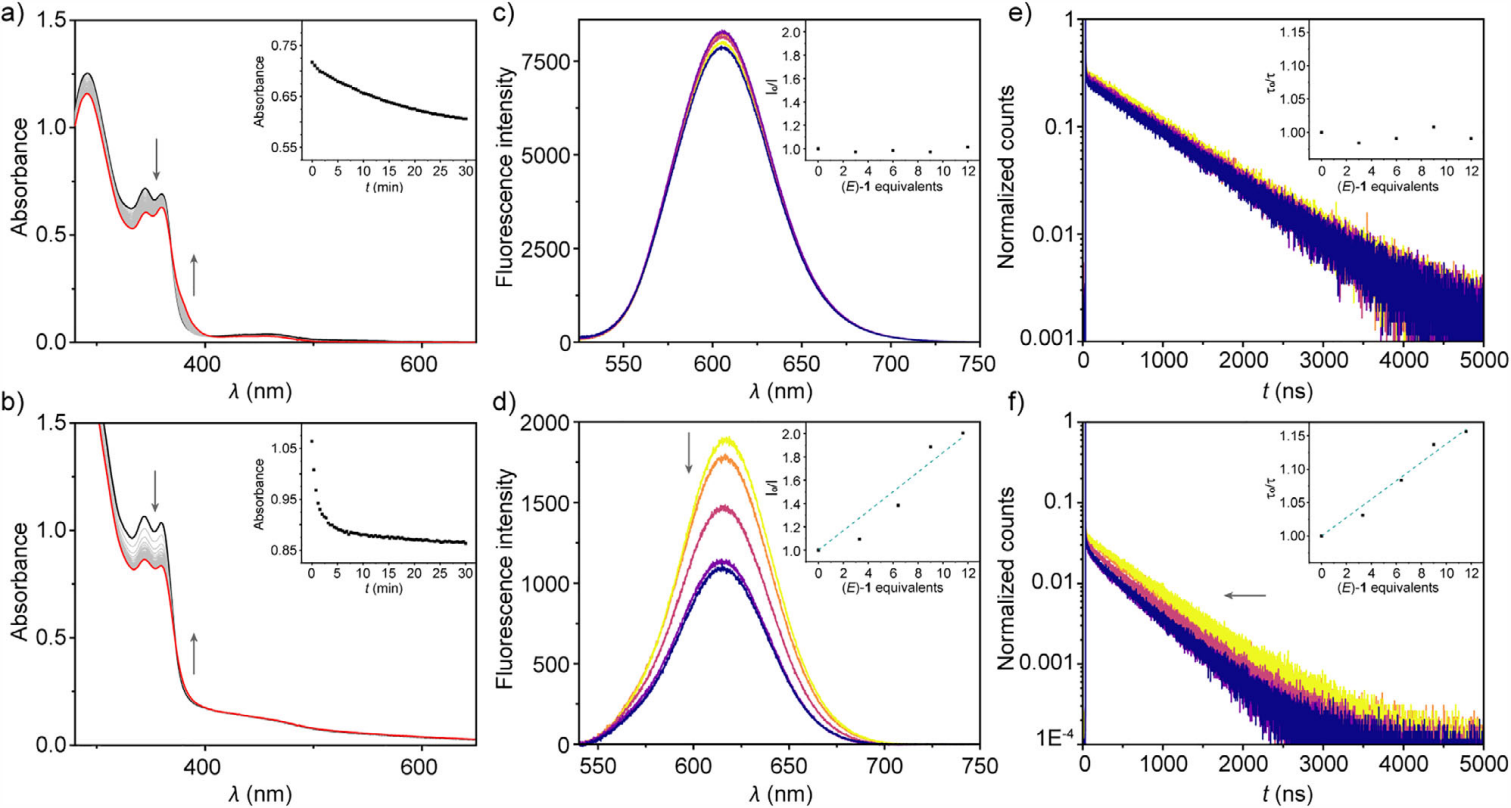
(a) UV–vis absorption spectral changes of (*E*)‐**1** (2.0 × 10^−5^ M) and [Ru(bpy)_2_(**L1**)]Cl_2_ (2.0 × 10^−6^ M) in DMSO upon irradiation with 455 nm light for 30 min; the inset shows the absorbance decrease at 345 nm as a function of time. (b) UV–vis absorption spectral changes of a 1 mM solution of lipid vesicles containing (*E*)‐**1** and [Ru(bpy)_2_(**L1**)]Cl_2_ (2.0 mol% and 0.2 mol% with respect to lipids, respectively) upon irradiation with 455 nm light; the inset shows the absorbance decrease at 345 nm as a function of time. c) Change of luminescence intensity of [Ru(bpy)_2_(**L1**)]Cl_2_ (1.0 µM) with increasing equivalents of (*E*)‐**1** in DMSO solution. d) Change of luminescence intensity of [Ru(bpy)_2_(**L1**)]Cl_2_ (0.7 µM) with increasing equivalents of (*E*)‐**1** in POPC‐LUVs. e) Luminescence decay profiles (logarithmic scale) of [Ru(bpy)_2_(**L1**)]Cl_2_ (1.0 µM) at 605 nm (*λ*_ex_ = 442 nm) with increasing equivalents of (*E*)‐**1** in DMSO solution. f) Luminescence decay profiles (logarithmic scale) of [Ru(bpy)_2_(**L1**)]Cl_2_ (0.7 µM) at 617 nm (*λ*_ex_ = 442 nm) with increasing equivalents of (*E*)‐**1** in POPC‐LUVs. The concentration of POPC was 0.50 mM.

Knowing that [Ru(bpy)_3_]Cl_2_ can sensitize the isomerization of stiff‐stilbene based transporter **1** in solution, we moved to a lipid bilayer environment. However, our attempts to incorporate [Ru(bpy)_3_]Cl_2_ in the bilayer of 1‐palmitoyl‐2‐oleoyl‐sn‐glycero‐3‐phosphocholine large unilamellar vesicles (POPC‐LUVs) were unsuccessful, due to the hydrophilicity of the ruthenium complex. The group of Bonnet [48, 49, 50] and others [51, 52, 53] have previously functionalized this complex with two alkyl tails to increase its lipophilicity, allowing membrane incorporation. These bis‐alkylated complexes were developed to perform photocatalytic reduction and oxidation reactions at the bilayer‐water interface. Hence, we selected for our studies the derivative [Ru(bpy)_2_(**L1**)]Cl_2_ where **L1** is a 2,2’‐bipyridine chelate functionalized with two C_17_H_35_ alkyl chains [49] (Scheme 1), and added 0.1 equiv. to a solution of (*E*)‐**1** in DMSO to see if its sensitization properties would be similar to those of parent [Ru(bpy)_3_]Cl_2_. Indeed, 455 nm irradiation of such a homogeneous mixture resulted in similar UV–vis and ^1^H NMR spectral changes as with [Ru(bpy)_3_]Cl_2_, indicating the formation of (*Z*)‐**1** (Figures 2a, S4, and S5). In such conditions, the PSS_PS_ (*E*/*Z*) ratio was changed to 73:27 using the alkylated bipyridyl complex, which is most likely due to somewhat diminished energy transfer to (*E*)‐**1** [43]. When the relative amount of [Ru(bpy)_2_(**L1**)]Cl_2_ was lowered to 0.01 equiv., virtually the same PSS_PS_ (*E*/*Z*) ratio was obtained, albeit that photoconversion was slower (Figures S6 and S7).

Next, both (*E*)‐**1** and [Ru(bpy)_2_(**L1**)]Cl_2_ were incorporated into the bilayer of POPC‐LUVs (2 mol% and 0.2 mol% with respect to lipids, respectively). The UV–vis spectrum of the aqueous liposome solution displayed the absorption bands of the stiff‐stilbene *E*‐isomer (*λ*_max_ = 344 and 360 nm) and of the ruthenium complex (MLCT band around *λ  *= 455 nm, Figure 2b). Upon irradiation with 455 nm light, the UV–vis spectral changes were similar as in DMSO solution, indicating that sensitized *E*→*Z* isomerization took place [54, 55]. It should be noted that the PSS_PS_ was reached quicker in the bilayer than in solution (see Figure 2a,b, insets), which hints at an improvement of photosensitization efficiency under membrane confinement.

Luminescence quenching as a result of energy transfer from [Ru(bpy)_2_(**L1**)]Cl_2_ to stiff‐stilbene based transporter **1** was then compared in solution and in the bilayer of POPC‐LUVs. The steady‐state emission of the ruthenium complex (*λ*_ex_ = 442 nm) exhibited a maximum at 605 nm in DMSO and at 617 nm in the bilayer (Figures 2c,d) [56]. Emission spectra were recorded in the presence of varying amounts of (*E*)‐**1** (0, 3, 6, 9, 12 equiv.), keeping the concentration of [Ru(bpy)_2_(**L1**)]Cl_2_ in either the DMSO or aqueous liposome solution constant (at 1.0 and 0.7 µM, respectively). For the DMSO solution, the emission showed only minimal variation under these conditions [57], while for the liposome solution the luminescence intensity decreased with increasing amount of (*E*)‐**1**, highlighting quenching of the ruthenium complex by the stiff‐stilbene photoswitch.

Time‐dependent luminescence decay profiles were then measured using the same DMSO and liposome samples to compare the efficiencies of the energy transfer process. The phosphorescence decay traces were analyzed using a biexponential (DMSO) or triexponential (liposome) fitting procedure. The lifetime of the longest decay component, attributed to tripletstate emission from the ruthenium complex, was shorter for [Ru(bpy)_2_(**L1**)]Cl_2_ in the liposome (598 ± 7 ns) than for [Ru(bpy)_2_(**L1**)]Cl_2_ in DMSO solution (866 ± 11 ns) [58]. Similar to the steady‐state experiments, in DMSO solution no significant quenching of this component was observed in presence of (*E*)‐**1** (Figure 2e and Table S1). In case of the liposome samples, on the contrary, the lifetime component assigned to [Ru(bpy)_2_(**L1**)]Cl_2_ triplet excited state emission decreased with increasing concentration of (*E*)‐**1** (Figure 2f and Table S2). A difference in Stern–Volmer slopes obtained in steady‐state and time‐resolved experiments (Figures S12 and S14) suggest a combination of dynamic and static quenching in the membrane.

**FIGURE 3:**
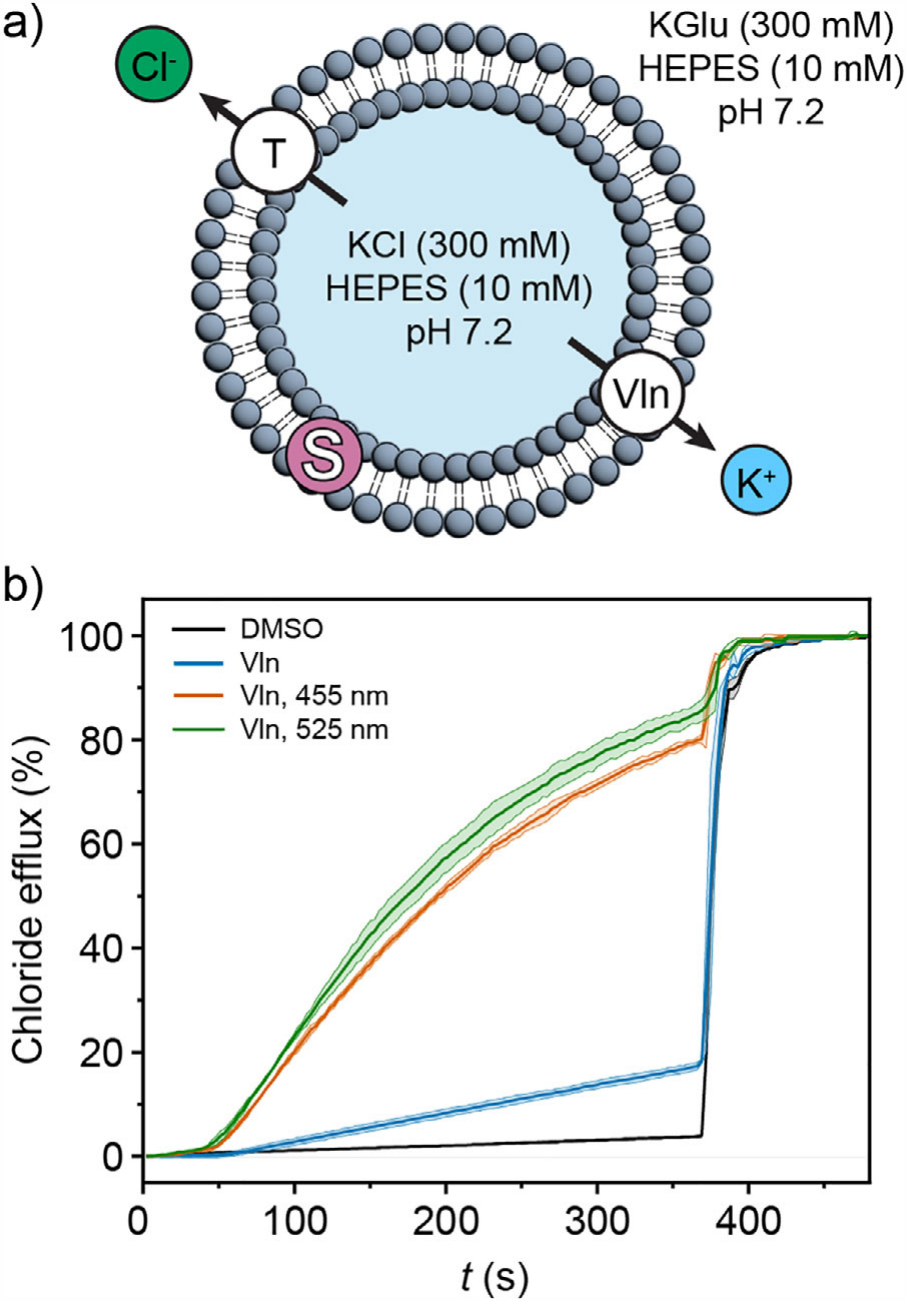
(a) Schematic drawing of the valinomycin cationophore‐coupled KCl efflux assay. (b) Chloride efflux from liposomes pre‐loaded with (*E*)‐**1** (2 mol%) and [Ru(bpy)_2_(**L1**)]Cl_2_ (0.2 mol%) upon the addition of valinomycin (0.1 mol% with respect to lipid) as co‐transporter of K^+^ cations, before (blue line) and after irradiation with 455 nm light for 1 min (orange line) or with 525 nm light for 5 min (green line). The control experiment (black line) where DMSO was added instead of a DMSO solution of valinomycin shows no chloride efflux.

Lastly, we conceived an experiment where this sensitization strategy can be used to activate transmembrane transport of chloride by visible light. Since the absorption and emission by the photosensitizer could interfere with commonly used fluorescence‐based transport assays, we opted for a cationophore‐coupled ion‐selective electrode (ISE) assay to monitor transport [59]. Here, the liposomes were loaded with KCl and suspended in potassium gluconate (KGlu), and both the internal and external aqueous phases were buffered to pH 7.2 using HEPES (Figure 3a). Previous mechanistic studies have shown that transporter (*Z*)‐**1**—being nearly 30 times more active than (*E*)‐**1**—is selective for Cl^−^ uniport over H^+^/Cl^−^ symport [11]. Therefore, valinomycin (Vln; a cyclic depsipeptide selective for K^+^ uniport) was used as co‐transporter to balance the charge. Addition of Vln to liposomes containing **1** initiated a net KCl efflux, which was followed with a chloride‐selective electrode in the external solution.

First, we tested whether the change in bilayer composition by incorporation of the photosensitizer affected the transport activity of (*Z*)‐**1**. Transport runs (performed in the dark) with and without [Ru(bpy)_2_(**L1**)]Cl_2_ (0.2 mol% with respect to lipids) in the membrane showed only a minimal difference in chloride efflux rate (Figure S16), meaning that the activity remained roughly the same. Also, (*E*)‐**1** still showed poor transport activity when the photosensitizer was added to the membrane (Figure 3b). To our delight, when the liposome sample containing (*E*)‐**1** and [Ru(bpy)_2_(**L1**)]Cl_2_ (2.0 mol% and 0.2 mol% with respect to lipids, respectively) was irradiated with 455 nm light for 60 s, either before or after Vln addition to initiate the transport experiment, a large increase of chloride efflux was observed (Figures 3b and S17). UV–vis spectral analysis after lysis of irradiated liposomes containing (*E*)‐**1** and [Ru(bpy)_2_(**L1**)]Cl_2_ confirmed that the more active form of the transporter, that is, (*Z*)‐**1**, was generated in the bilayer (Figure S23) and hence, that the observed increase in chloride efflux was due to photosensitized *E*→*Z* isomerization. Importantly, irradiation for the same time of liposomes containing (*E*)‐**1** (and not the sensitizer) led to only a negligible increase in transport (Figure S18) [54]. Furthermore, irradiation of POPC‐LUVs containing only [Ru(bpy)_2_(**L1**)]Cl_2_ did not result in measurable chloride efflux, neither at 10‐times higher loading (2.0 mol% with respect to lipids, see Figures S20–S22). This result excluded photoinduced membrane leakage (e.g. by lipid oxidation) [60, 61]. Moreover, at the used [Ru(bpy)_2_(**L1**)]Cl_2_ loading, no singlet oxygen could be detected in the liposome solution (Figures S24–S28).

Lastly, owing to the broad MLCT absorption band of the ruthenium complex, activation of transport could also be achieved using 525 nm light, although a longer irradiation time (5 min) was needed (Figure 2b). With this longer wavelength, no transport enhancement was observed in the absence of the photosensitizer (Figure S19).

## 3 Conclusions

We have achieved sensitized isomerization of stiff‐stilbene in solution and the lipid bilayer using a lipophilic ruthenium(II) polypyridyl complexes. The confinement of the sensitizer and the photoswitch within the bilayer enhances energy transfer due to the increase in local concentrations. This sensitization strategy was applied to activate transmembrane chloride transport by a light‐switchable transporter, where now visible light (455 or 525 nm) could be used instead of UV light (365 nm). We expect our approach to be applicable also to other photoresponsive membrane‐embedded (supra)molecular systems, avoiding the need for using UV light, which can be damaging to materials and cells. Further, we anticipate that asymmetric insertion of the photosensitizer in the bilayer allows predominant *E*→*Z* isomerization at one side of the membrane, which would give rise to active transport (against a chloride gradient) [46, 47].

## Conflicts of Interest

The authors declare no conflicts of interest.

## Supporting information




**Supporting File 1**: anie72049‐sup‐0001‐SuppMat.Pdf.

## Data Availability

The data that support the findings of this study are available in the supporting information of this article.
