# Supplementary material for: Visible‐Light Sensitized Isomerization in the Lipid Bilayer Enables Activation of a Transmembrane Transporter
Source: Angew Chem Int Ed Engl. 2026 Apr 5;65(20):e6015167. doi: 10.1002/anie.6015167 (PMC13159413; doi:10.1002/anie.6015167)
Supplement: Supplementary file 1 — Supporting File 1: anie72049‐sup‐0001‐SuppMat.Pdf. [file ANIE-65-e6015167-s001.pdf]

SUPPORTING INFORMATION

Visible-Light Sensitized Isomerization in the Lipid Bilayer Enables Activation of a Transmembrane Transporter

Julia Villalva, Abhishek Mondal, Willem Marulanda, Jasper E. Bos,

Sylvestre Bonnet, Anjali Pandit, and Sander J. Wezenberg*

Leiden Institute of Chemistry, Leiden University,

Einsteinweg 55, 2333 CC Leiden, The Netherlands

Email: s.j.wezenberg@lic.leidenuniv.nl

Table of contents

1. General methods and materials S2
2. ^{1}H NMR irradiation studies S3
3. UV-Vis irradiation studies S7
4. Luminescence quenching experiments S9
5. Transport experiments S15
6. Singlet oxygen generation studies S22
7. References S27

1. General methods and materials

DMSO-d_6 was purchased from Eurisotop and stored under N_2 over molecular sieves (4Å). Degassing of solvents was carried out by purging with Ar for 10 min, unless noted otherwise. Compounds (E)-**1**,[1] (*Z*)-**1**,[1] and [Ru(bpy)$_2$(**L1**)]Cl_2[2] were prepared using procedures reported in the literature. [Ru(bpy)$_3$]Cl_2 was purchased from Sigma-Aldrich and used without further purification. ^{1}H NMR spectra were recorded on a Bruker 500 Ultra Shield instrument at 298 K. UV-Vis spectra were recorded on an Agilent Cary 8454 spectrometer using 1 cm quartz cuvettes. The kinetic data shown in Figures 2a-b, S6, S7 and S8 was obtained by monitoring the same sample at different time intervals. Luminescence was measured on a JASCO FP-8500 spectro-fluorimeter using 1 cm quartz cuvettes. Time-resolved fluorescence experiments were performed on a FluoTime 300 Spectrometer (PicoQuant, Berlin) using a 442 nm pulsed diode laser as irradiation source with a repetition rate of 0.2 MHz. Samples were measured at 20 °C using 1 cm quartz cuvettes. The instrument response function (IRF; ~40 ps full width half maximum) was measured using the scatter signal of a solution of Ludox® colloidal silica. DLS was measured on a Malvern Zetasizer Nano S instrument using BRAND UV-cuvettes micro. Irradiation of samples was carried out using Thorlabs model M455F3 (24.5 mW) and LED525E (2.6 mW) LEDs positioned at a distance of 1 cm to the sample. The Thorlabs model M455F3 (24.5 mW) irradiation source was used in combination with a 435 nm cut-on filter. The chloride concentrations during the cationophore-coupled transport assay were determined using an Accumet chloride-selective electrode.

Liposome preparation

A lipid film of a mixture of POPC and compound (*E*)-**1** or (*Z*)-**1** (2 mol% or 0.5 mol% with respect to POPC, respectively) and/or [Ru(bpy)$_2$(**L1**)]Cl_2 (0.2 mol% with respect to POPC) was prepared from a THF solution under reduced pressure and then dried under vacuum overnight. The lipid film was rehydrated by vortexing with the internal solution consisting of potassium chloride (KCl, 300 mM) buffered to pH 7.2 with HEPES (10 mM). The lipid suspension was then subjected to 9 freeze-thaw cycles and left to rest at room temperature for 30 minutes. After this, the suspension was extruded 25 times through a 200 nm polycarbonate membrane. The vesicles were then passed through a Sephadex® column in order to remove non-incorporated (*E*)-**1**, (*Z*)-**1** and [Ru(bpy)$_2$(**L1**)]Cl_2 and to exchange the external solution for one containing potassium gluconate (KGlu, 300 mM) buffered to pH 7.2 with HEPES (10 mM).

Note: The procedure was carried out in the dark, except for the preparation of the lipid film. Although the THF solution was protected from ambient light, slight sensitization could have taken place.

2. ¹H NMR irradiation studies

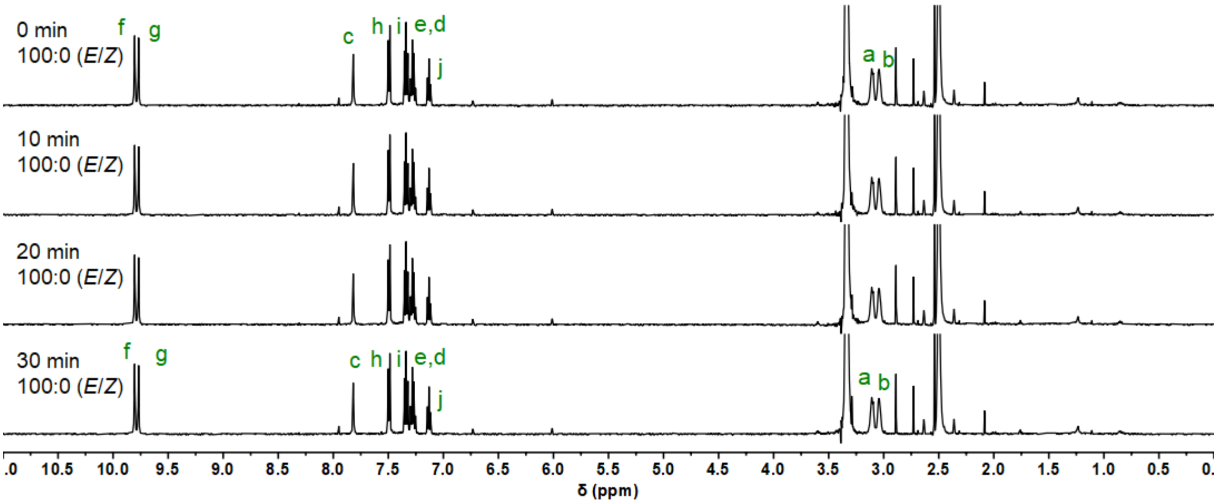

Figure S1. ^{1}H NMR spectral changes (500 MHz, 293 K) of (*E*)-**1** (2.0 mM) in DMSO-d_6 upon irradiation with 455 nm light for 30 min. See Scheme 1 in main document for atom labels.

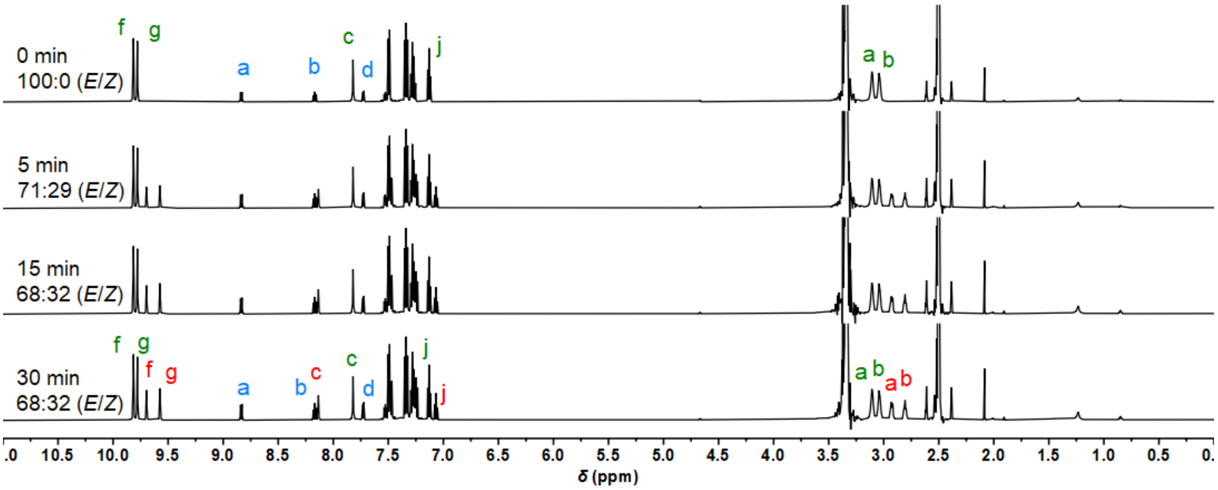

Figure S2. ^{1}H NMR spectral changes (500 MHz, 293 K) of (*E*)-**1** (2.0 mM) and $[Ru(bpy)_3]Cl_2$ (0.20 mM) in DMSO-d_6 upon irradiation with 455 nm light for 30 min. The (*E*/*Z*) ratios were calculated by averaging the integrals of the thiourea NH (f and g), and aromatic H (c and j) signals.

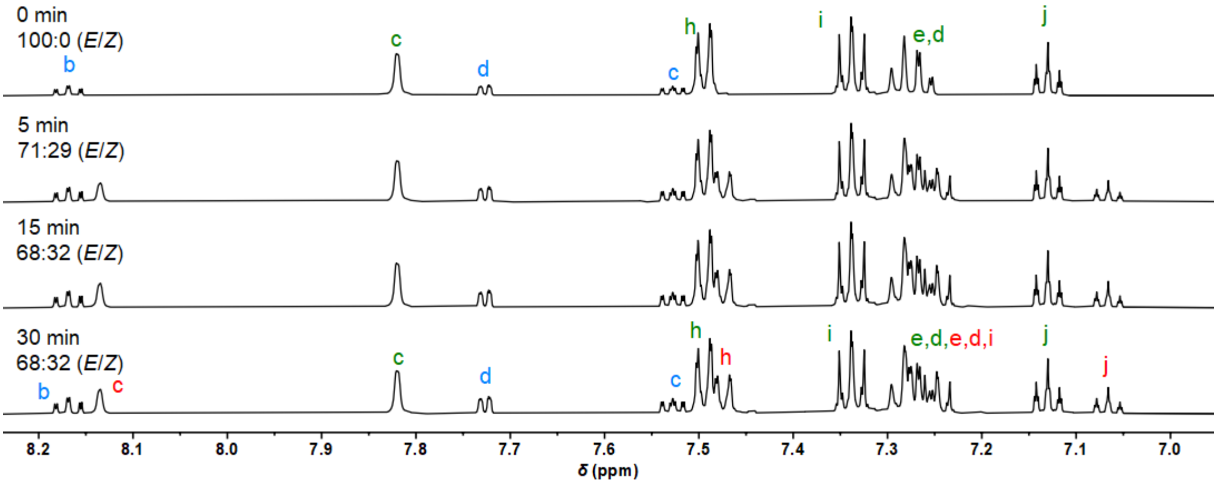

Figure S3. Selected aromatic region (7.0-8.2 ppm) region of the ^{1}H NMR spectrum shown in Figure S2.

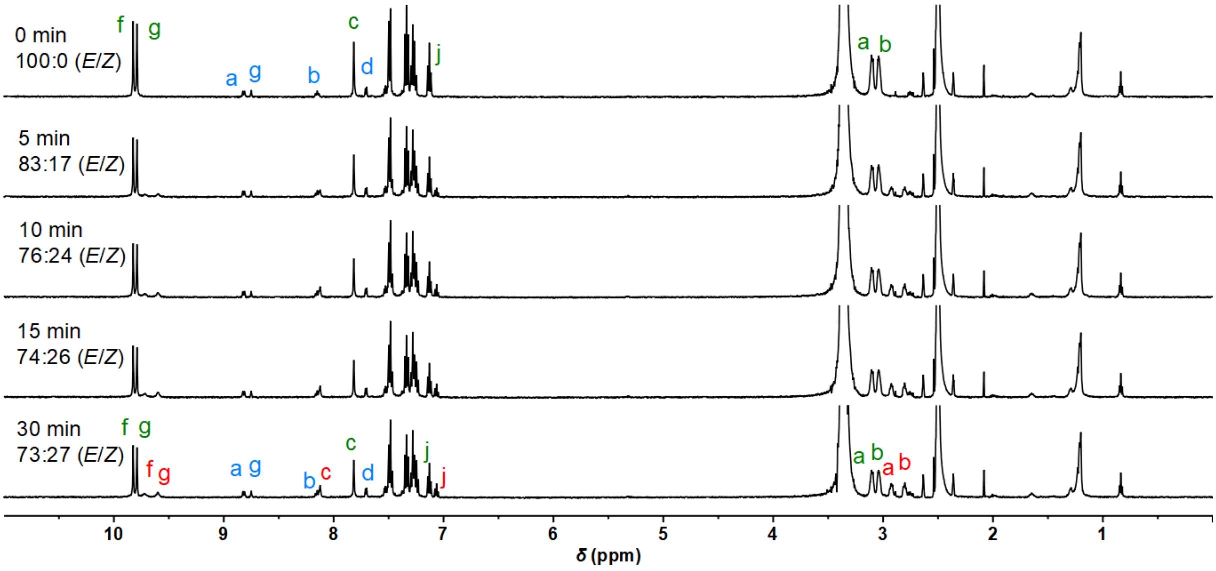

Figure S4. ^{1}H NMR spectral changes (500 MHz, 293 K) of (*E*)-**1** (2.0 mM) and [Ru(bpy)$_2$(**L1**)]Cl$_2$ (0.20 mM) upon irradiation with 455 nm light for 30 min. The (*E*/*Z*) ratios were calculated using the integrals of the aromatic H (j) signals.

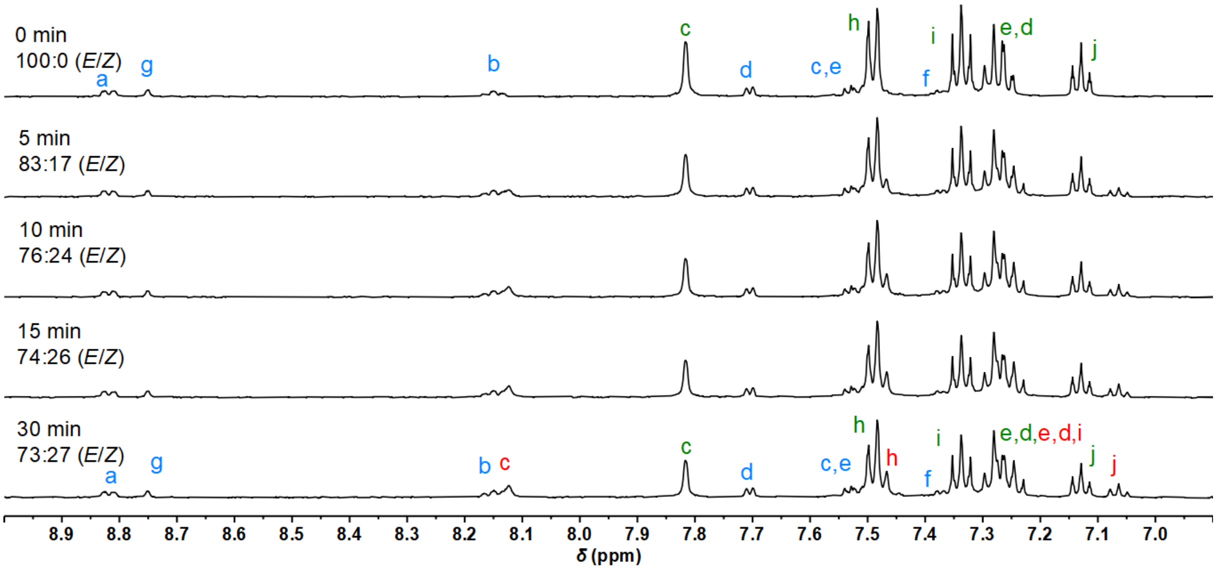

Figure S5. Selected aromatic region (6.9-9.0 ppm) of the ^{1}H NMR spectrum shown in Figure S4.

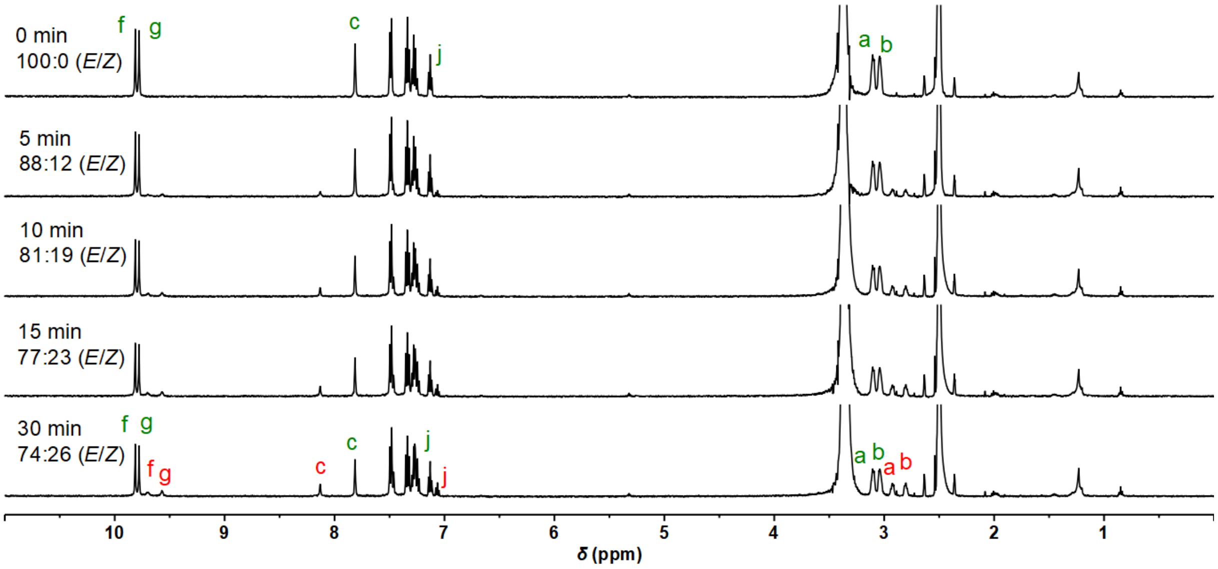

Figure S6. ^{1}H NMR spectral changes (500 MHz, 293 K) of (*E*)-**1** (2.0 mM) and [Ru(bpy)$_2$(**L1**)]Cl$_2$ (0.02 mM) upon irradiation with 455 nm light for 30 min. The (*E*/*Z*) ratios were calculated by averaging the integrals of the aromatic H (c and j) signals.

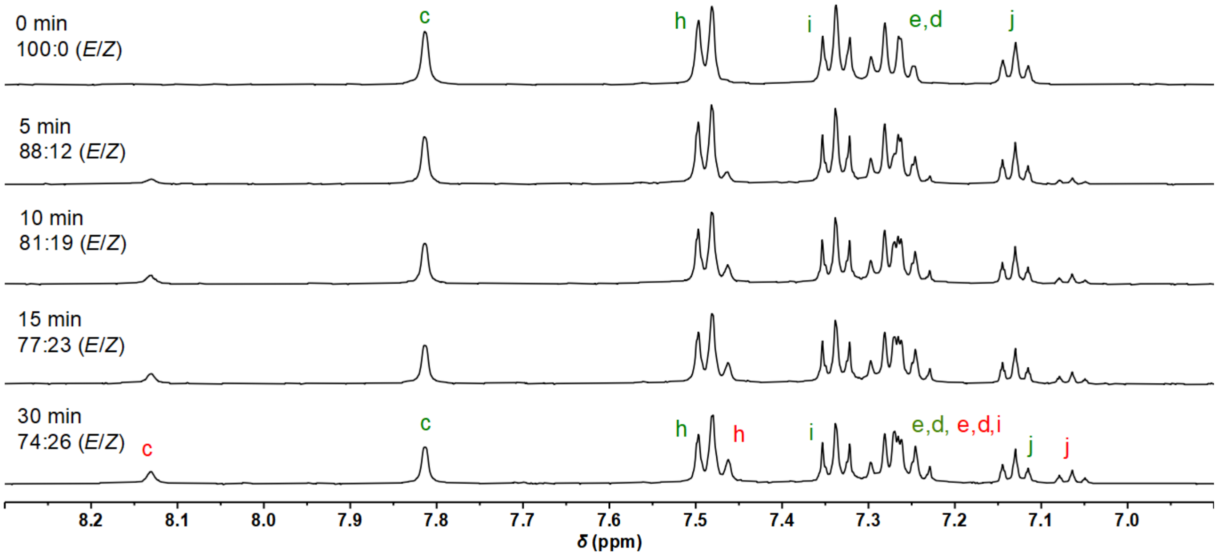

Figure S7. Selected aromatic region (6.9-9.0 ppm) of the ^{1}H NMR spectrum shown in Figure S5.

3. UV-Vis irradiation studies

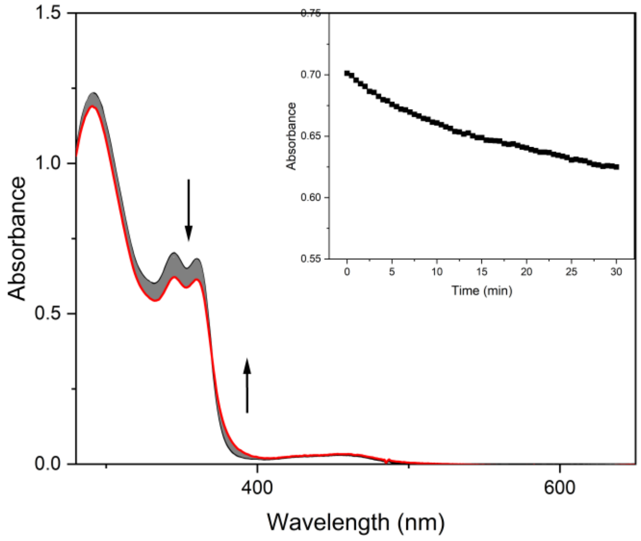

Figure S8. UV-Vis spectral changes of a solution of (*E*)-**1** ($2.0×10^{-5}$ M) and $[Ru(bpy)_3]Cl_2$ ($2.0×10^{-6}$ M) in dry and degassed DMSO upon irradiation with 455 nm light for 30 min.

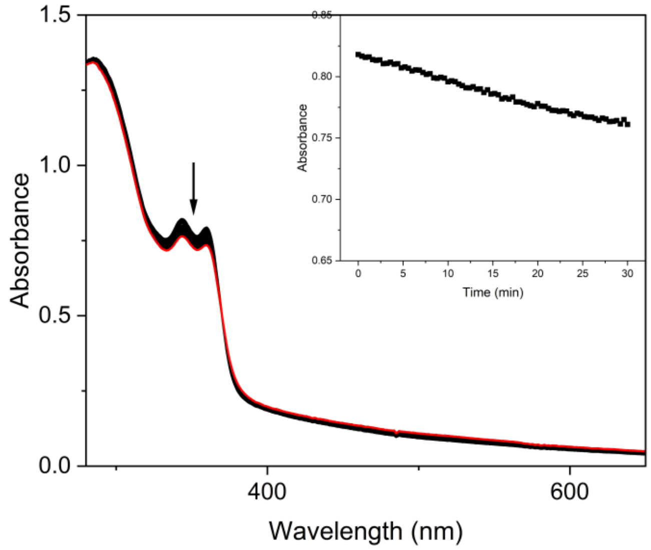

Figure S9. UV-Vis spectral changes of a 1.0 mM solution of lipid vesicles containing 2 mol% (*E*)-**1** upon irradiation with 455 nm light for 30 min.

In order to follow potential changes in the [Ru(bpy)$_2$(**L1**)]Cl$_2$ absorption upon 455 nm light irradiation, its loading was increased from 0.2 mol% to 2 mol% with respect to the lipid content. The photostability of the complex was studied upon irradiation with 455 nm light under Argon. After 50 min irradiation no photodissociation or alternative photodegradation reactions could be detected.

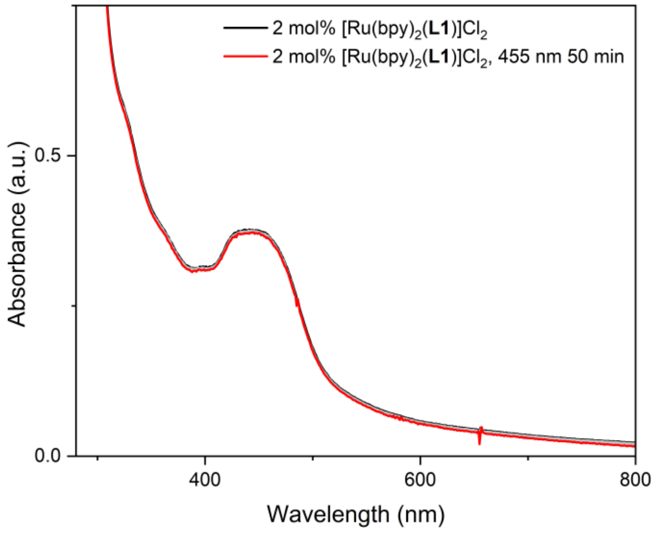

Figure S10. UV-Vis spectral changes of a 1.0 mM solution of lipid vesicles containing [Ru(bpy)$_2$(**L1**)]Cl$_2$ 2 mol% (black line) upon irradiation with 455 nm light for 50 min (red line).

4. Luminescence quenching experiments

4.1. Conditions for the experiments:

Steady-State Luminescence Measurements:

Steady-state luminescence spectra were recorded using an excitation wavelength of 442 nm. Emission was monitored at 605/650 nm in DMSO and 617/650 nm in liposome environments. Spectral scans were conducted at a rate of 200 nm/s with a step size of 0.1 nm. Samples were degassed by bubbling argon for 20 minutes to minimize oxygen interference. The emission intensity was compared after 10 and 20 minutes of degassing to confirm completion of the degassing procedure.

Time-Resolved Luminescence Measurements:

Time-resolved luminescence decay measurements were carried out using the same excitation wavelength of 442 nm. Emission detection was set at 605 or 650 nm in DMSO and 617 or 650 nm in the liposomes. Each measurement was performed over a 10-minute period within a 0–5 μs time range. Samples were degassed by bubbling argon for 20 minutes to minimize oxygen interference. Emission decay curves were analysed according to a multi-exponential fitting procedure, following deconvolution of the IRF, using the software FluoFit (PicoQuant, Berlin).

4.2. Steady state experiments

In order to minimize inner filter effects, the $[Ru(bpy)_2(\mathbf{L1})]Cl_2$ concentration was kept at around 1 μM. In sections 4.2.1 and 4.2.2, the results obtained for the steady-state experiments are shown for both DMSO and liposome samples. At these low photosensitizer concentrations, background contributions from the Raman scatter and minor impurities to the emission signal are significant, therefore, a background correction was performed in all samples, subtracting the signal of a blank solution with DMSO. The results before and after background subtraction are shown below. It is worth mentioning that the background correction does not play a significant role in the changes in the relative intensity of the $[Ru(bpy)_2(\mathbf{L1})]Cl_2$ luminescence with respect to the (*E*)-**1** equivalents.

The relative emission intensity of $[Ru(bpy)_2(\mathbf{L1})]Cl_2$ was measured at two different points: the maximum of the emission peak (605 nm in DMSO or 617 nm in liposomes) and 650 nm, where the contribution of the background before its subtraction was minimal. The results at both wavelengths point to the same conclusions.

4.2.1. DMSO solution

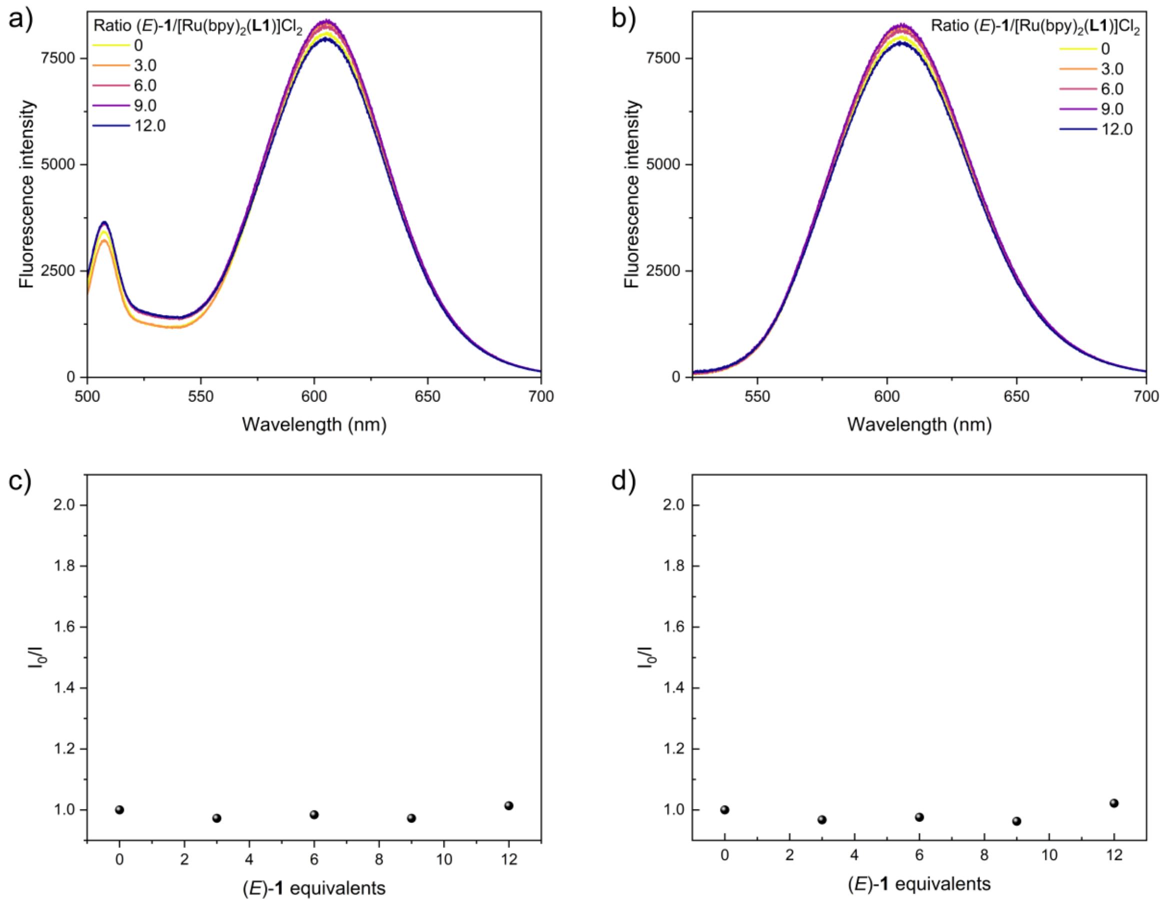

Figure S11. a) Change of luminescence intensity of $[Ru(bpy)_2(\mathbf{L1})]Cl_2$ with increasing concentration of (*E*)-**1** in DMSO solutions. b) Change of luminescence intensity of $[Ru(bpy)_2(\mathbf{L1})]Cl_2$ with increasing concentration of (*E*)-**1** in DMSO solutions after DMSO background subtraction. I_0/I vs (*E*)-**1** equivalents plots obtained from the data shown in (b) using the intensity of $[Ru(bpy)_2(\mathbf{L1})]Cl_2$ emission at (c) 605 nm and (d) 650 nm. Experimental conditions: $[Ru(bpy)_2(\mathbf{L1})]Cl_2$ conc. = 1.0 μM. Excitation wavelength was fixed at 442 nm.

4.2.2. Lipid bilayer

For the liposome samples, a new emission band appeared when the concentration of (*E*)-**1** increased (centered at around 520 nm). We ascribe this band to emission from (*E*)-**1** aggregates, present in the bilayer due to the high local concentration.

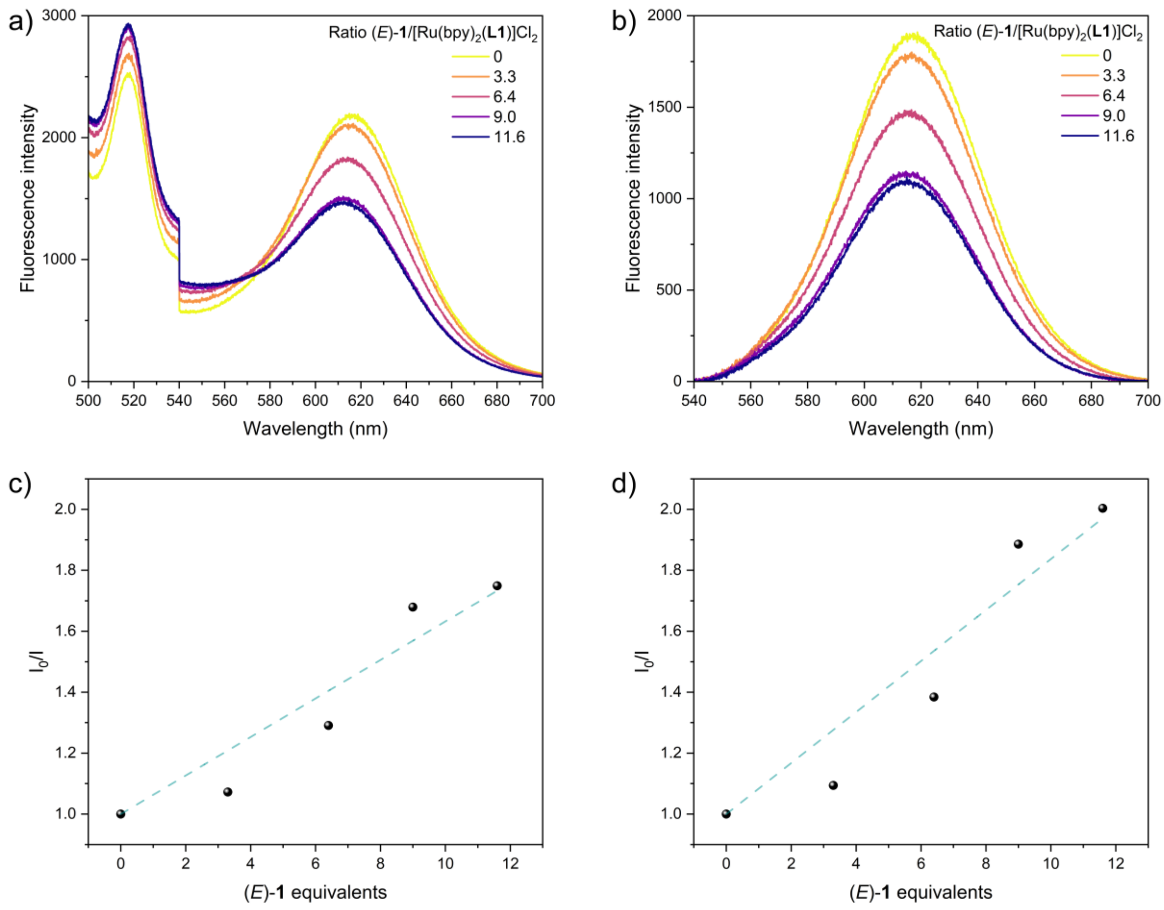

Figure S12. a) Change of luminescence intensity of $[Ru(bpy)_2($**L1**$)]Cl_2$ with increasing concentration of (*E*)-**1** in POPC vesicles. b) Change of luminescence intensity of $[Ru(bpy)_2($**L1**$)]Cl_2$ with increasing concentration of (*E*)-**1** in POPC vesicles after removal of the vesicles contribution to the luminescence spectra. Stern−Volmer plots obtained from the data shown in (b) using the intensity of $[Ru(bpy)_2($**L1**$)]Cl_2$ emission at (c) 617 nm, slope = 0.063 or (d) 650 nm, slope = 0.084. Experimental conditions: $[Ru(bpy)_2($**L1**$)]Cl_2$ conc. = 0.7 μM, POPC conc. = 0.5 mM. Excitation wavelength was fixed at 442 nm.

4.3. Time resolved experiments

In order to compare the time-resolved and steady-state experiments, the $[Ru(bpy)_2(\mathbf{L1})]Cl_2$ emission lifetime was also measured at the maximum of the emission peak (605 nm in DMSO and 617 nm in the liposome) and at 650 nm, for both DMSO and liposome samples.

4.3.1. Decay profiles in DMSO solution at 605 and 650 nm

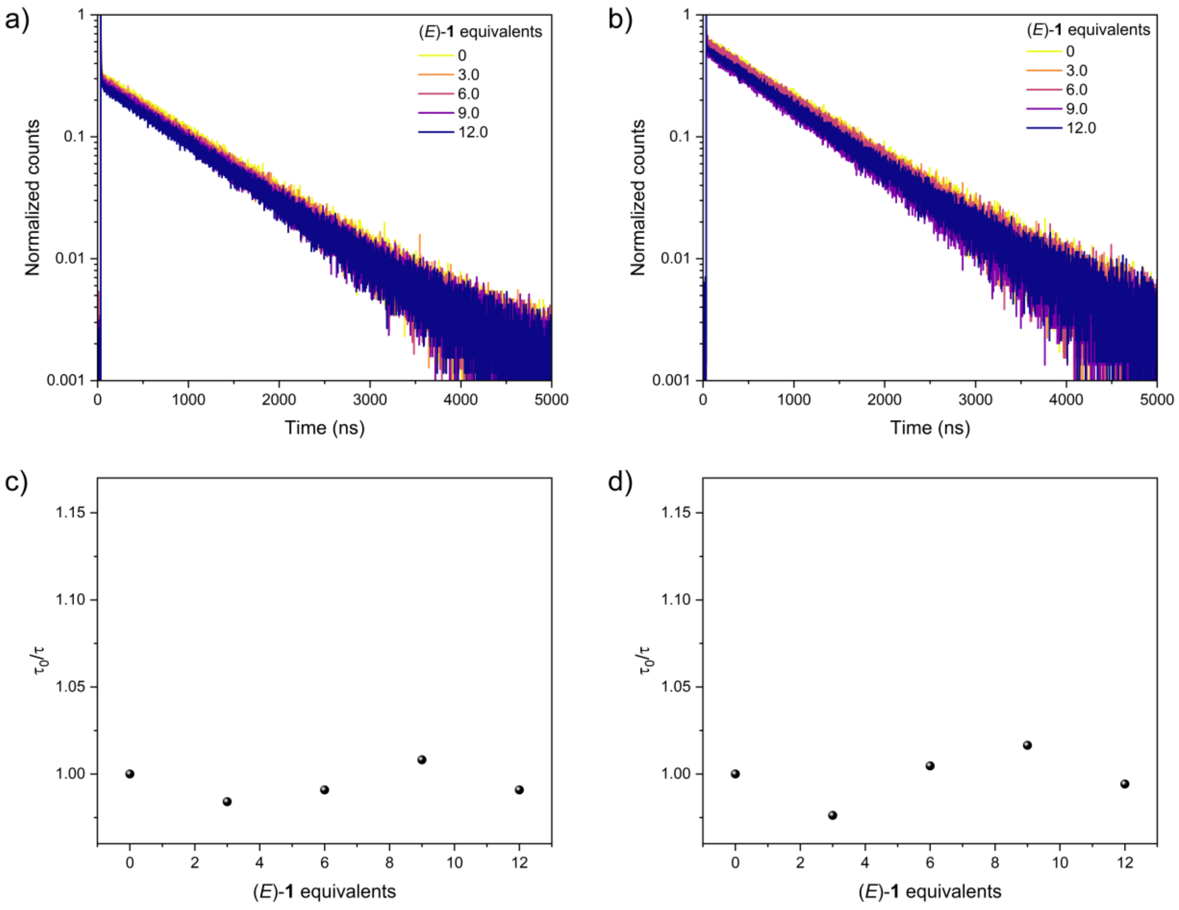

Figure S13. Luminescence decay profiles (logarithmic scale) of $[Ru(bpy)_2(\mathbf{L1})]Cl_2$ at (a) 605 nm and (b) 650 nm upon increasing concentrations of (*E*)-**1** in dry DMSO solution. c) τ_0/τ vs (*E*)-**1** equivalents from the data shown in (a). d) τ_0/τ vs (*E*)-**1** equivalents from the data shown in (b). Experimental conditions: $[Ru(bpy)_2(\mathbf{L1})]Cl_2$ conc. = 1.0 μM. Excitation wavelength was fixed at 442 nm.

4.3.2. Decay profiles in the lipid bilayer at 617 and 650 nm

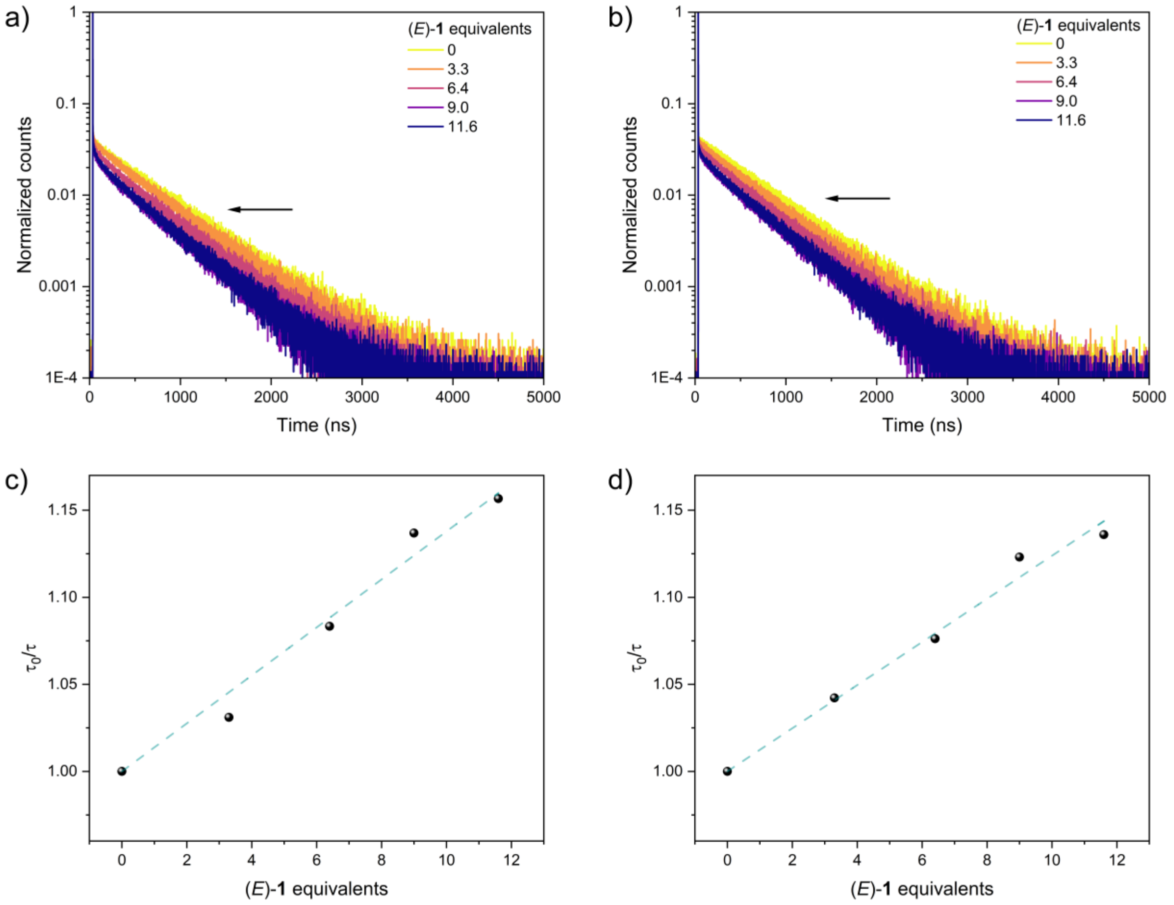

Figure S14. Luminescence decay profiles (logarithmic scale) of [Ru(bpy)$_2$(**L1**)]Cl$_2$ at (a) 617 nm and (b) 650 nm upon increasing concentrations of (*E*)-**1** in POPC vesicles. c) Stern−Volmer plot obtained from the data shown in (a), slope = 0.014. d) Stern−Volmer plot obtained from the data shown in (b), slope = 0.012. Experimental conditions: POPC conc. = 0.5 mM, [Ru(bpy)$_2$(**L1**)]Cl$_2$ conc. = 0.7 μM. Excitation wavelength was fixed at 442 nm.

4.4. Fitting of time-resolved data

4.4.1. DMSO Solution

The curves were fitted to two lifetime components (Figure 2e). The shortest one (τ_2) was only noticeable at low concentrations of [Ru(boy)2(**L1**)]Cl_2 and therefore ascribed to solvent impurities. The long decay (τ_1) was attributed to triplet state emission from the ruthenium complex.

	605 nm		650nm	
	τ_1/ns (a_1 %)	τ_2/ns (a_2 %)	τ_1/ns (a_1 %)	τ_2/ns (a_2 %)
[Ru(bpy)$_2$(**L1**)]Cl_2	866±11 (35.5)	4±1 (64.5)	863±10 (61.9)	3±3 (38.1)
(*E*)-**1**/[Ru(bpy)$_2$(**L1**)]Cl_2 (3.0)	880±10 (39.6)	4±1 (60.4)	884±10 (53.0)	4±3 (47.1)
(*E*)-**1**/[Ru(bpy)$_2$(**L1**)]Cl_2 (6.0)	874±9 (24.2)	3.2±0.4 (75.6)	859±9 (54.6)	3±2 (45.4)
(*E*)-**1**/[Ru(bpy)$_2$(**L1**)]Cl_2 (9.0)	859±9 (22.4)	3.1±0.3 (77.6)	849±9 (45.0)	3±1 (55.0)
(*E*)-**1**/[Ru(bpy)$_2$(**L1**)]Cl_2 (12.0)	874±9 (30.3)	3.6±0.9 (69.7)	868±10 (40.7)	2.4±0.5 (59.3)

Table S1. Time-resolved photoluminescence data in dry DMSO. λ_{exc} = 442 nm, λ_{em} = 605 nm or λ_{em} = 650 nm. [Ru(bpy)$_2$(**L1**)]Cl_2 conc. = 1.0 μM.

4.4.2. Lipid bilayer

In the lipid bilayer, the presence of (*E*)-**1** required consideration of a third decay component (τ_3), which is tentatively ascribed to emission from aggregates of (*E*)-**1**.

	617 nm			650 nm		
	τ_1/ns (a_1 %)	τ_2/ns (a_2 %)	τ_3/ns (a_3 %)	τ_1/ns (a_1 %)	τ_2/ns (a_2 %)	τ_3/ns (a_3 %)
[Ru(bpy)$_2$(**L1**)]Cl_2	598±7 (59.9)	5±3 (40.1)		593±8 (92.3)	3±2 (7.8)	
(*E*)-**1**/[Ru(bpy)$_2$(**L1**)]Cl_2 (3.3)	580±7 (25.6)	2±0.5(68.4)	50±20 (6.0)	569±6 (69.0)	3±3 (25.8)	93±80 (5.2)
(*E*)-**1**/[Ru(bpy)$_2$(**L1**)]Cl_2 (6.4)	552±7 (20.5)	1.7±0.4 (72.3)	65±16 (7.2)	551±6 (61.3)	4±3 (28.5)	103±36 (10.2)
(*E*)-**1**/[Ru(bpy)$_2$(**L1**)]Cl_2 (9.0)	526±7 (20.3)	2.3±0.5 (70.6)	74±14 (9.1)	528±6 (50.7)	4±2 (36.2)	105±25 (13.1)
(*E*)-**1**/[Ru(bpy)$_2$(**L1**)]Cl_2 (11.6)	517±7 (16.4)	1.8±0.4 (74.5)	59±10 (8.7)	522±6 (45.2)	3±2 (42.1)	88±21 (12.8)

Table S2. Time-resolved photoluminescence data in 0.5 mM POPC lipid bilayer vesicles. λ_{exc} = 442 nm, λ_{em} = 617 nm or λ_{em} = 650 nm. [Ru(bpy)$_2$(**L1**)]Cl_2 conc. = 0.7 μM as determined by UV-Vis. Molar ratio [Ru(bpy)$_2$(**L1**)]Cl_2/POPC = 7:5000.

5. Transport experiments

ISE assay[3,4]

For each measurement, the lipid solutions were diluted with the external buffered solution to a standard volume (5.0 mL) with a lipid concentration of 1.0 mM. The valinomycin cationophore was added as a DMSO solution (1 µM, 0.1 mol%) to start the experiment (t = 60 s). After 6 min, detergent (50 µL of Triton X-100 (11 wt%) in H_2O:DMSO (7:1 v/v)) was added to lyse the vesicles and the 100% chloride efflux reading was taken at 8 min.

The used Accumet chloride-selective electrode was calibrated against aqueous NaCl solutions of known concentrations prior to each experiment according to the supplier's manual. A calibration curve was generated by fitting to the Nerst equation (Equation S1).

$$y = a + b \log x \quad (\mathbf{S1})$$

Where *y* is the potential (mV), *x* is the chloride concentration and *a* and *b* are the parameters to be fitted. Using the calibration curve, the readings (mV) of the experiment were converted to chloride concentrations and subsequently to percentages of efflux according to equation S2.

$$Efflux = \frac{C_t - C_0}{C_d - C_0} \times 100 \quad (\mathbf{S2})$$

Where C_t is the concentration at time t, C_0 is the initial concentration, measured at t = 3 s, and C_d is the final concentration, measured at t = 480 s.

All the experiments were performed in the dark. For the irradiation experiments, the solution was first purged with Argon for 10 min. DLS measurements confirmed that the vesicle structure was retained during the bubbling process and that the size distribution of the vesicles was not altered (Figure S13).

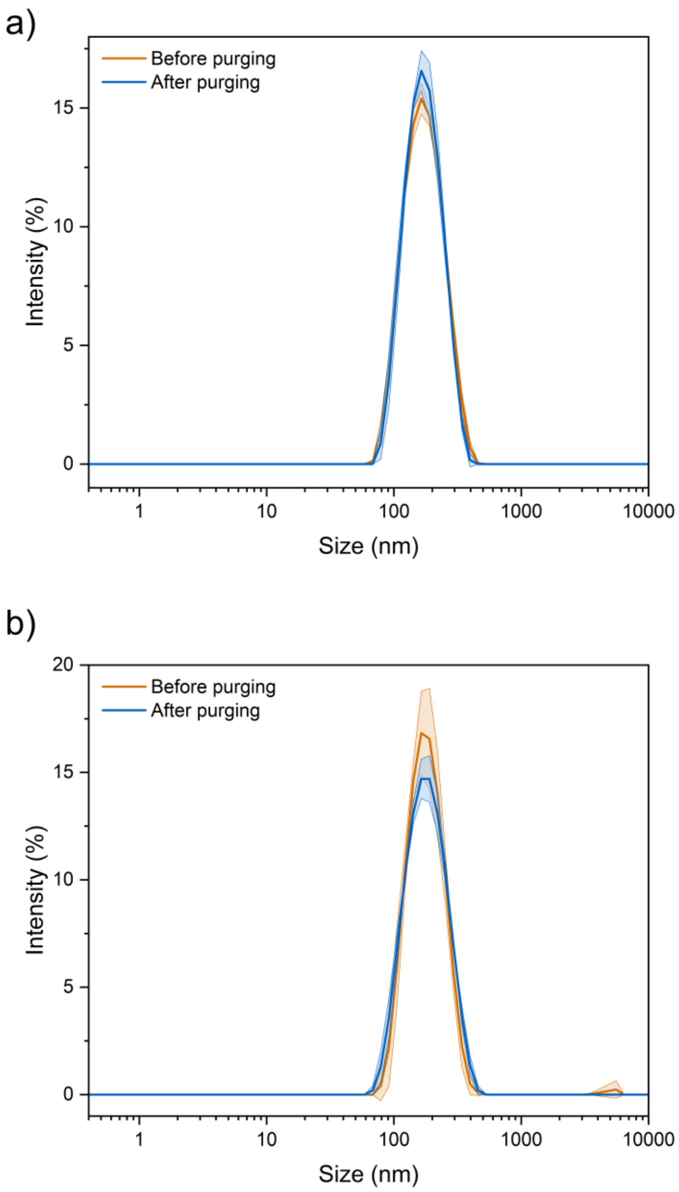

Figure S15. Size distribution report by intensity of POPC liposomes under purging conditions in a) vesicles loaded with a mixture of (*E*)-**1** (2 mol%) and [Ru(bpy)$_2$(**L1**)]Cl$_2$ (0.2 mol%) and b) vesicles loaded with (*E*)-**1** (2 mol%).

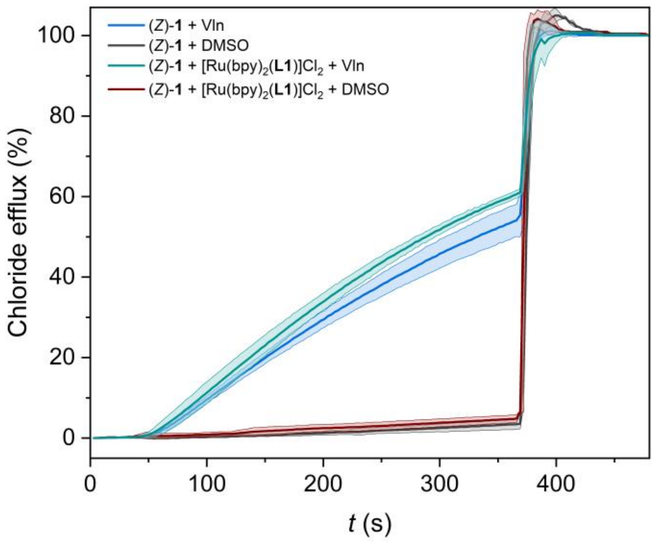

Figure S16. Cationophore-coupled assays in liposomes containing (*Z*)-**1** (0.5 mol% with respect to lipid) or a mixture of (*Z*)-**1** (0.5 mol% with respect to lipid) and [Ru(bpy)$_2$(**L1**)]Cl$_2$ (0.2 mol% with respect to lipid). Each point represents the average of 2 repeats.

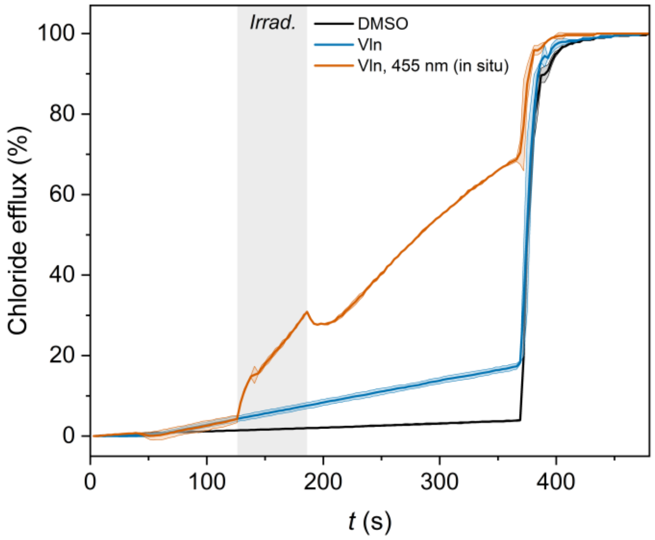

Figure S17. Chloride efflux mediated by (*E*)-**1** (2 mol%) in presence of [Ru(bpy)$_2$(**L1**)]Cl$_2$ (0.2 mol%) and valinomycin (0.1 mol% with respect to lipid), where 455 nm irradiation is performed *in-situ* from 126-186 s. The black line corresponds to the control where DMSO is added instead of valinomycin. Although exposure of the AgCl electrode to 455 nm light leads to a change in electrical potential during irradiation, the expected values are recovered once the irradiation stops.

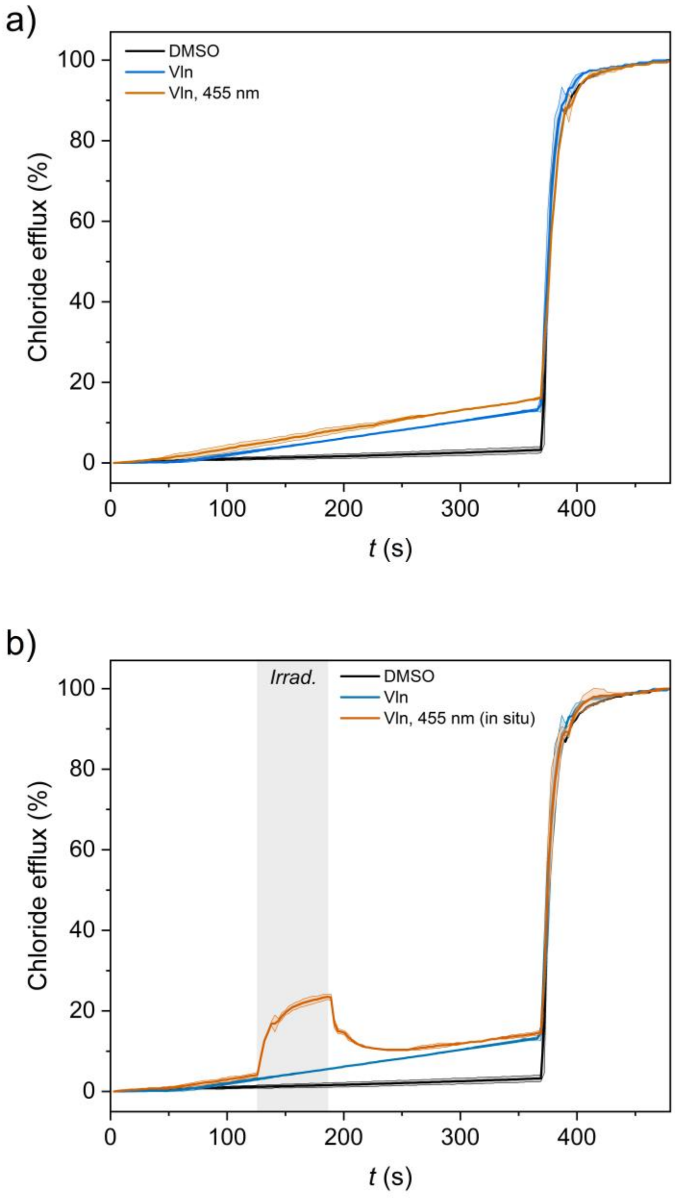

Figure S18. a) Chloride efflux mediated by (*E*)-**1** (2 mol%) in the presence of valinomycin (0.1 mol% with respect to lipid) without (blue line) and with (orange line) irradiation with 455 nm light for 1 min before valinomycin is added. The black line corresponds to the control where DMSO is added instead of valinomycin. b) Chloride efflux mediated by (*E*)-**1** (2 mol%) in the presence of valinomycin (0.1 mol% with respect to lipid), where 455 nm irradiation is performed *in-situ* from 126-186 s. Although exposure of the AgCl electrode to 455 nm light leads to a change in electrical potential during irradiation (126-186 s), the expected values are recovered once the irradiation stops.

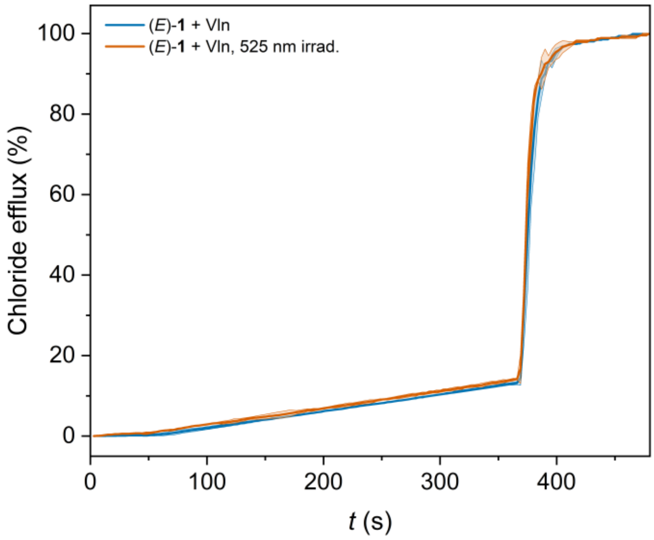

Figure S19. Chloride efflux mediated by (*E*)-**1** (2 mol%) in the presence of valinomycin (0.1 mol% with respect to lipid) without (blue line) and with (orange line) irradiation with 525 nm light for 5 min before valinomycin is added.

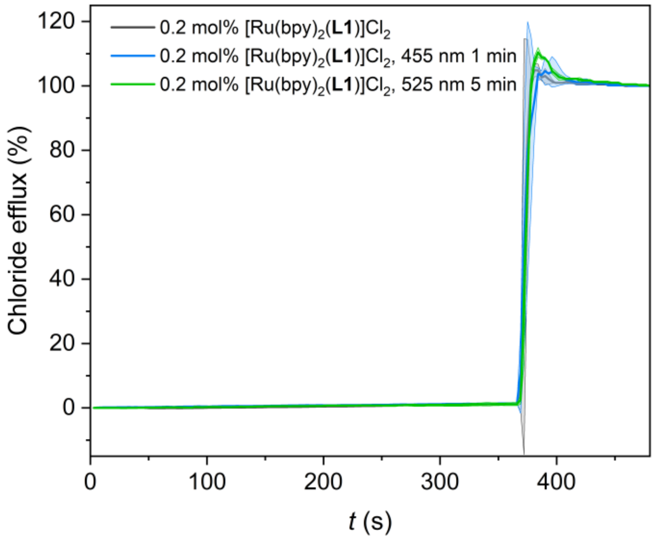

Figure S20. Cationophore-coupled assays using liposomes containing only $[Ru(bpy)_2($**L1**$)]Cl_2$ (0.2 mol% with respect to lipid). Irradiation conditions: 455 nm (1 min) or 525 nm (5 min) before valinomycin addition. Each point represents the average of 2 repeats.

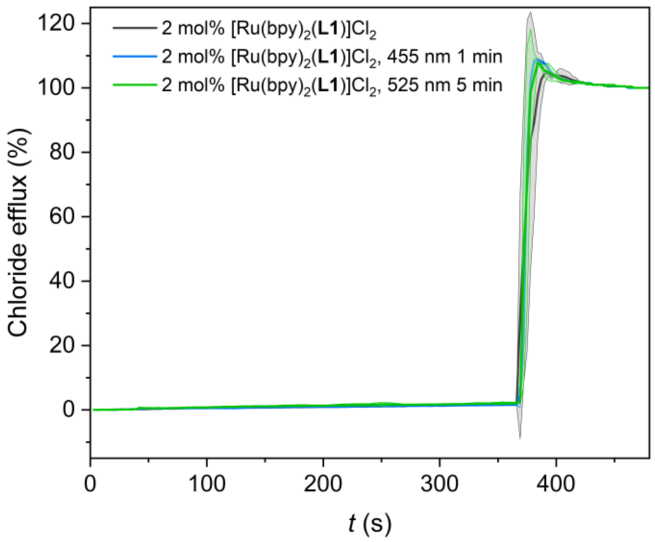

Figure S21. Cationophore-coupled assays using liposomes containing only $[Ru(bpy)_2($**L1**$)]Cl_2$ (2 mol% with respect to lipid). Irradiation conditions: 455 nm (1 min) or 525 nm (5 min) before valinomycin addition. Each point represents the average of 2 repeats.

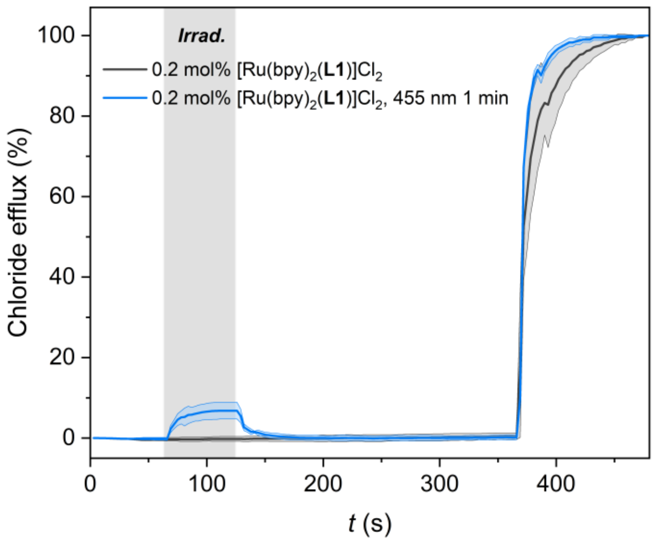

Figure S22. Cationophore-coupled assays using liposomes containing only $[Ru(bpy)_2($**L1**$)]Cl_2$ (0.2 mol% with respect to lipid). Irradiation conditions: 455 nm (1 min) *in-situ* after valinomycin addition. Each point represents the average of 2 repeats.

One batch of liposomes containing (*E*)-**1** (2 mol%) and [Ru(bpy)$_2$(**L1**)]Cl$_2$ (0.2 mol%) that was used in the cationophore-coupled chloride transport experiments (main text, Figure 3b) was analyzed before and after the transport experiment in order to confirm *E*→*Z* isomerization. For this, detergent (25 µL of Triton X-100 (11 wt%) in H_2O:DMSO (7:1 v/v)) was added to 2.5 mL of the original 1 mM liposome solution before irradiation. The absorbance of the obtained solution was consistent with the presence of both (*E*)-**1** and [Ru(bpy)$_2$(**L1**)]Cl$_2$ (grey line in Figure S21). UV-Vis analysis of the irradiated liposomes obtained after transport experiments (where the lysing step is already performed as part of the assay) showed absorbance changes that confirm *E*→*Z* isomerization (orange line in Figure S23).

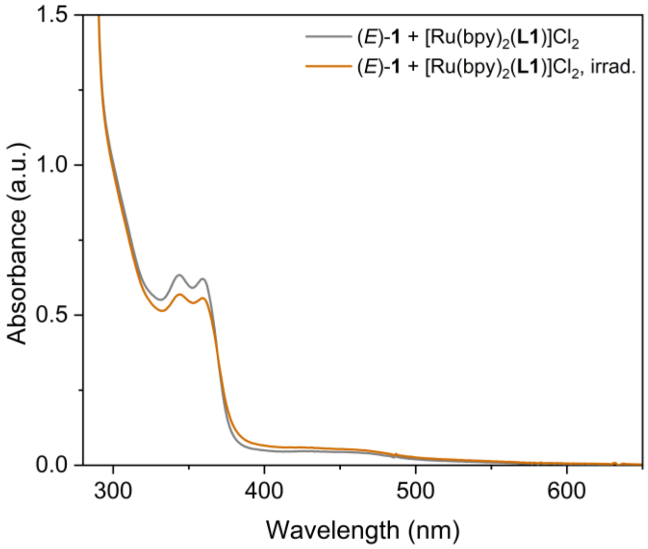

Figure S23. UV-Vis spectra of lysed solutions of 1 mM lipid vesicles containing (*E*)-**1** 2 mol% and [Ru(bpy)$_2$(**L1**)]Cl$_2$ 0.2 mol% before (grey line) and after (orange line) irradiation with 455 nm light.

6. Singlet oxygen generation studies

Potential generation of singlet oxygen upon excitation of the [Ru(bpy)$_2$(**L1**)]Cl$_2$ photosensitizer was evaluated following a reported protocol.[5] As a singlet oxygen trapping agent, 9,10-anthracenedipropanoic acid (ABDA) was used, which exhibits an absorption band at 378 nm. In presence of $^1O_2$, ABDA forms an endoperoxide, causing a loss of conjugation and consequently a decrease in 378 nm absorbance.

To establish the baseline response of the singlet oxygen probe under the assay conditions, control experiments were performed in the absence of photosensitizer to verify that irradiation of lipid vesicles alone does not cause a measurable loss of ABDA absorbance. Large unilamellar vesicles (LUVs) were prepared following the same procedure and using the same buffer composition and conditions as employed for the ion-selective electrode (ISE) transport assays (total lipid concentration = 1.0 mM). ABDA (100 µM) was added to the external aqueous solution. UV–Vis spectra were recorded at 10 s intervals over a total period of 100 s under two conditions: (i) in the dark for 100 s and (ii) irradiation with a 455 nm LED for 100 s, using the same irradiation setup as used in the activation experiments.

No change in the ABDA absorption at 378 nm was observed in either the dark control or during 455 nm irradiation, with the spectra fully overlapping throughout the measurement (Figure S24). The result confirms that under the assay conditions, lipid vesicles and light exposure alone do not induce ABDA photobleaching or detectable singlet oxygen formation.

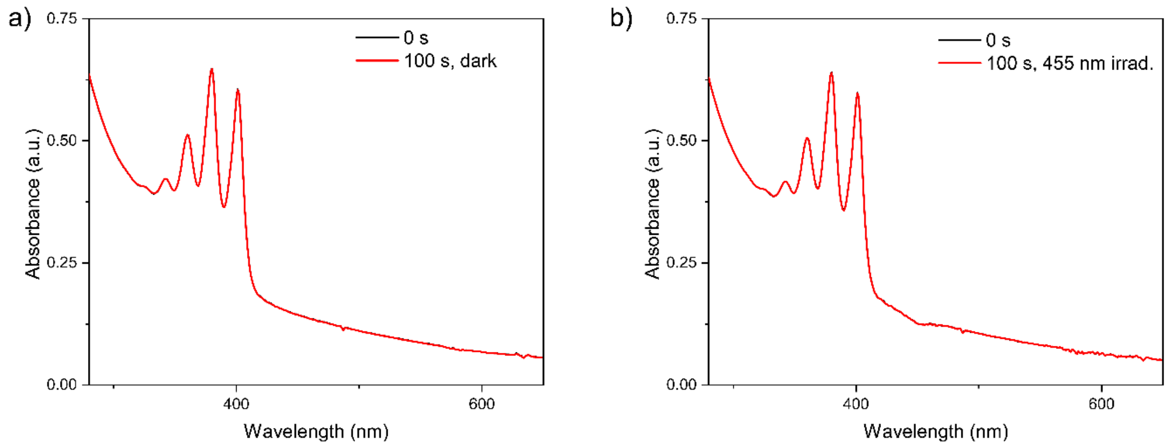

Figure S24. UV-Vis spectral changes of a 1.0 mM solution of lipid vesicles, with ABDA (100 µM) present in the external solution upon (a) 100 s standing in the dark and (b) irradiation with 455 nm light for 100 s. The spectra were taken at 10 s intervals.

To evaluate whether the photosensitizer generates singlet oxygen under the conditions required for transporter activation, singlet oxygen trapping experiments were performed with lipid vesicles containing pre-incorporated [Ru(bpy)$_2$(**L1**)]Cl$_2$ (20 µM). Importantly, the concentration of the photosensitizer was identical to that used in the photochemical activation experiments. ABDA (100 µM) was added to the

external aqueous solution to monitor potential $^{1}O_2$ formation in the system. UV–Vis absorption spectra were recorded at 10 s intervals in the dark for 100 s and under continuous irradiation with a 455 nm LED for 100 s.

No significant change in the ABDA absorption at 378 nm was observed in either dark or during 455 nm irradiation (Figure S25). The result indicates that, at the photosensitizer concentration required for transporter activation, no significant singlet oxygen is generated under the applied experimental conditions.

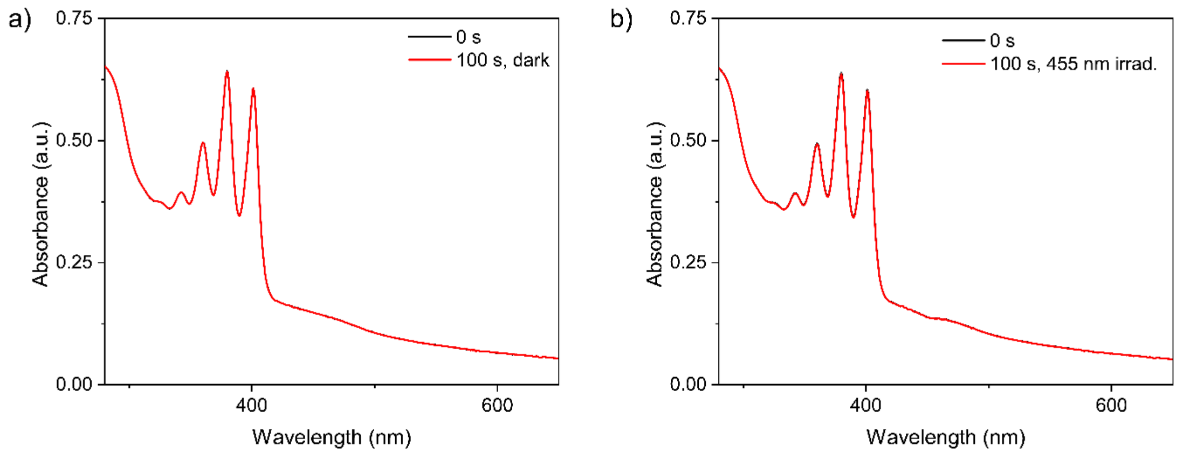

Figure S25. UV-Vis spectral changes of a 1.0 mM solution of lipid vesicles containing pre-incorporated [Ru(bpy)$_2$(**L1**)]Cl$_2$ (20 µM), with ABDA (100 µM) present in the external solution upon (a) 100 s standing in the dark and (b) irradiation with 455 nm light for 100 s. The spectra were taken at 10 s intervals.

Experiments were further performed in the presence of both the photosensitizer and the (*E*)-**1** transporter using identical conditions. [Ru(bpy)$_2$(**L1**)]Cl$_2$ (20 µM) and the (*E*)-**1** (200 µM) were co-incorporated into the lipid bilayer. ABDA (100 µM) was added to the external aqueous solution. UV–Vis absorption spectra were recorded at 10 s intervals in the dark for 100 s and under continuous irradiation with a 455 nm LED for 100 s.

Upon light irradiation, pronounced spectral changes were observed corresponding to the photoisomerization of (*E*)-**1** to (*Z*)-**1**. In contrast, the characteristic ABDA absorption band at 378 nm remained unchanged throughout the irradiation period. (Figure S26) The absence of any detectable decrease in ABDA absorbance, despite efficient photoisomerization of the transporter, indicates that no significant singlet oxygen is generated under the experimental conditions and sensitizer concentration required for transporter activation.

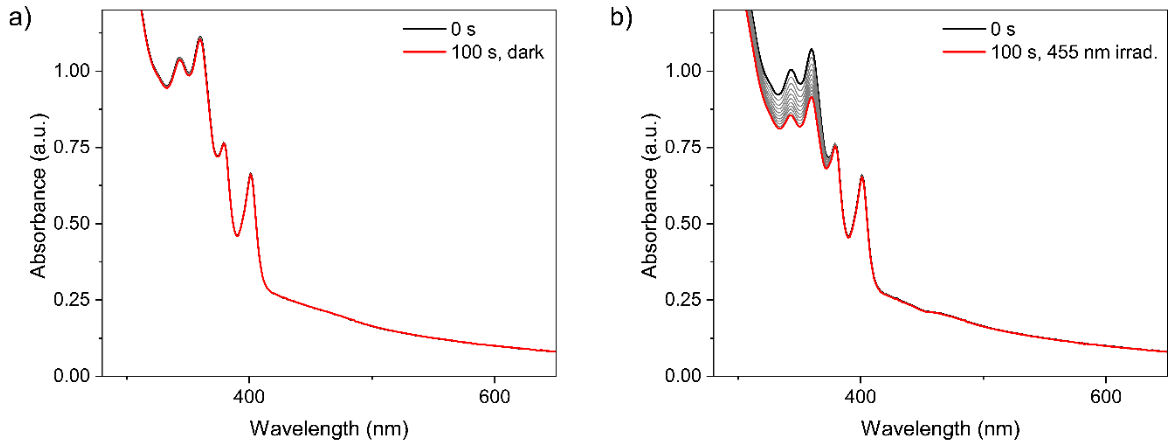

Figure S26. UV-Vis spectral changes of a 1.0 mM solution of lipid vesicles containing pre-incorporated [Ru(bpy)$_2$(**L1**)]Cl$_2$ (20 µM) and (*E*)-**1** (200 µM), with ABDA (100 µM) present in the external solution upon (a) 100 s standing in the dark and (b) irradiation with 455 nm light for 100 s. The spectra were taken at 10 s intervals.

To validate the sensitivity of the singlet oxygen trapping assay control experiments were performed using the water-soluble photosensitizer [Ru(bpy)$_3$]Cl$_2$ in the absence of lipid vesicles and transporter.

Experiments were carried out in the same buffer solution as used for the liposome-based assays. ABDA (100 µM) was added to the buffer, and [Ru(bpy)$_3$]Cl$_2$ was added at final concentrations of either 20 µM or 50 µM. UV–Vis absorption spectra were recorded at 10 s intervals in the dark for 100 s and under continuous irradiation with a 455 nm LED for 100 s.

At a sensitizer concentration of 20 µM, no change in the ABDA absorption band at 378 nm was observed either in the dark or upon light irradiation, with the spectra fully overlapping throughout the measurement. In contrast, when the sensitizer concentration was increased to 50 µM, a gradual decrease in the ABDA absorption at 378 nm was observed during irradiation, consistent with singlet oxygen generation and subsequent ABDA endoperoxide formation (Figure S27). These results confirm that the ABDA assay is sufficiently sensitive to detect singlet oxygen under the applied experimental conditions and demonstrate that singlet oxygen generation by ruthenium bipyridyl complexes becomes significant only at sensitizer concentrations higher than those employed for transporter activation.

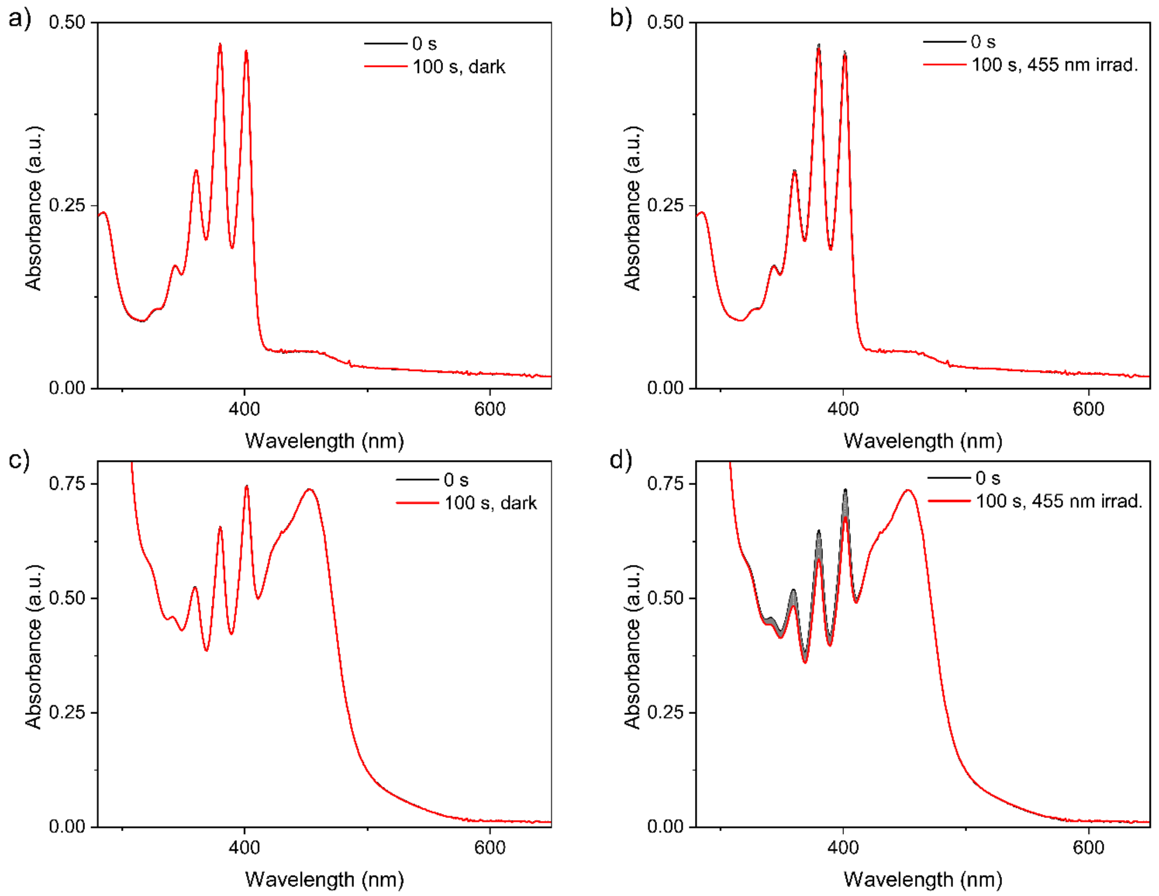

Figure S27. UV-Vis spectral changes of a 1.0 mM solution of lipid vesicles, with ABDA (100 μM) and $[Ru(bpy)_3]Cl_2$ (20 μM) (a and b) or $[Ru(bpy)_3]Cl_2$ (50 μM) (c and d) present in the external solution upon (a and c) 100 s standing in the dark and (b and d) irradiation with 455 nm light for 100 s. The spectra were taken at 10 s intervals.

Lastly, experiments were performed under oxygen-enriched conditions by purging the system with molecular oxygen. Buffer solutions containing ABDA (100 μM) and $[Ru(bpy)_3]Cl_2$ at final concentrations of either 20 μM or 50 μM were prepared as described above. Prior to spectral measurements, each solution was purged with molecular oxygen for 5 minutes to increase the dissolved oxygen concentration. UV–Vis absorption spectra were recorded at 10 s intervals in the dark for 100 s and under continuous irradiation with a 455 nm LED for 100 s.

Under oxygen-enriched conditions, no detectable change in the ABDA absorption band at 378 nm was observed at a sensitizer concentration of 20 μM, either in the dark or upon irradiation. In contrast, for samples containing 50 μM $[Ru(bpy)_3]Cl_2$, a markedly faster decrease in ABDA absorbance was observed during irradiation (Figure S28) compared to non-purged samples (Figure S27), consistent with enhanced singlet oxygen production in the presence of elevated molecular oxygen levels.

These observations further corroborate that singlet oxygen generation by ruthenium bipyridyl complexes is both sensitizer-concentration-dependent and oxygen-concentration-dependent, and that under the

experimental conditions and sensitizer loading used for transporter activation (20 μM), no significant singlet oxygen generation occurs.

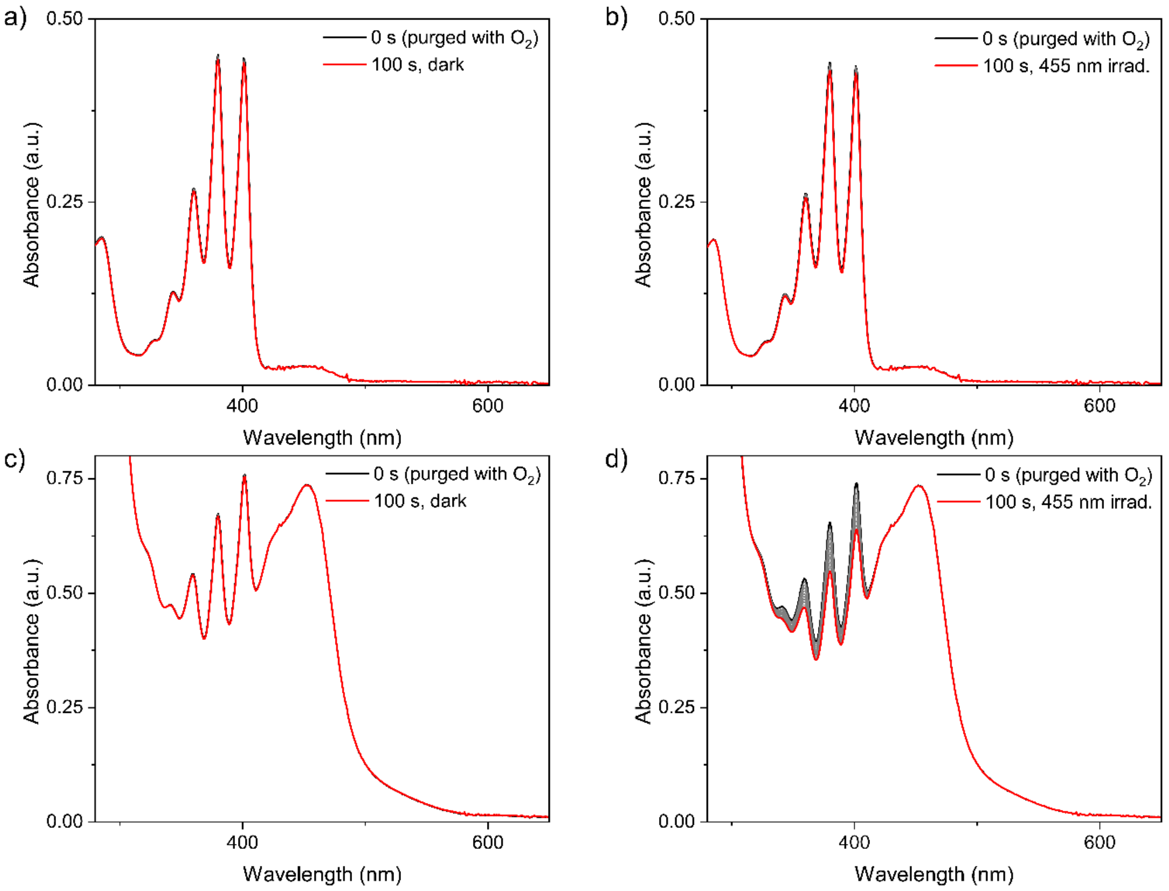

Figure S28. UV-Vis spectral changes of a 1.0 mM solution of lipid vesicles, with ABDA (100 μM) and $[Ru(bpy)_3]Cl_2$ (20 μM) (a and b) or $[Ru(bpy)_3]Cl_2$ (50 μM) (c and d) present in the external solution upon (a and c) 100 s standing and (b and d) irradiation with 455 nm light for 100 s. The spectra were taken at 10 s intervals.

7. References

[1] S. J. Wezenberg, B. L. Feringa, *Nat. Commun.* **2018**, *9*, 1984.

[2] H. Song, A. Amati, A. Pannwitz, S. Bonnet, L. Hammarström, *J. Am. Chem. Soc.* **2022**, *144*, 19353-19364.

[3] X. Wu, L. W. Judd, E. N. W. Howe, A. M. Withecombe, V. Soto-Cerrato, H. Li, N. Busschaert, H. Valkenier, R. Pérez-Tomás, D. N. Sheppard, Y.-B. Jiang, A. P. Davis, P. A. Gale, *Chem* **2016**, *1*, 127-146.

[4] X. Wu, E. N. W. Howe, P. A. Gale, *Acc. Chem. Res.* **2018**, *51*, 1870-1879.

[5] X.-Q. Zhou, M. Xiao, V. Ramu, J. Hilgendorf, X. Li, P. Papadopoulou, M. A. Siegler, A. Kros, W. Sun, S. Bonnet, *J. Am. Chem. Soc.* **2020**, *142*, 10383-10399.
